# Neuromodulatory co-expression in cardiac vagal motor neurons of the dorsal motor nucleus of the vagus

**DOI:** 10.1016/j.isci.2024.110549

**Published:** 2024-07-19

**Authors:** Eden Hornung, Shaina Robbins, Ankita Srivastava, Sirisha Achanta, Jin Chen, Zixi Jack Cheng, James Schwaber, Rajanikanth Vadigepalli

**Affiliations:** 1 Daniel Baugh Institute of Functional Genomics/Computational Biology, Department of Pathology and Genomic Medicine, Thomas Jefferson University, Philadelphia, PA 19107, USA; 2 Burnett School of Biomedical Sciences, College of Medicine, University of Central Florida, BMS Building 20, Room 230, 4110 Libra Drive, Orlando, FL 32816, USA

**Keywords:** Molecular neuroscience, Cellular neuroscience, Omics, Bioengineering

## Abstract

Vagal innervation is well known to be crucial to the maintenance of cardiac health, and to protect and recover the heart from injury. Only recently has this role been shown to depend on the activity of the underappreciated dorsal motor nucleus of the vagus (DMV). By combining neural tracing, transcriptomics, and anatomical mapping in male and female Sprague-Dawley rats, we characterize cardiac-specific neuronal phenotypes in the DMV. We find that the DMV cardiac-projecting neurons differentially express pituitary adenylate cyclase-activating polypeptide (PACAP), cocaine- and amphetamine-regulated transcript (CART), and synucleins, as well as evidence that they participate in neuromodulatory co-expression involving catecholamines. The significance of these findings is enhanced by previous knowledge of the role of PACAP at the heart and of the other neuromodulators in peripheral vagal targets.

## Highlights

• Unique spatial transcriptomic profile of the dorsal motor nucleus of the vagus (DMV)
• Distinctive neuromodulatory co-expression defines cardiac-projecting DMV neurons
• Cardiac-projecting DMV neurons co-express cholinergic and catecholaminergic markers
• Left intermediate DMV cardiac-projecting neurons express PACAP, synuclein, and CART

## Graphical abstract

**Figure:**
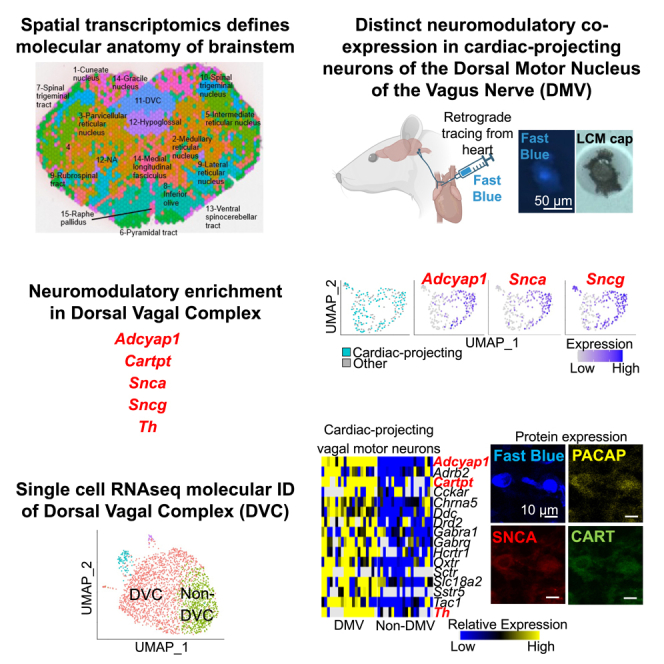

## Introduction

During the development of heart failure, hemodynamic abnormalities result in neurohormonal activation, which causes autonomic imbalance by a relative increase in sympathetic activity and withdrawal of vagal activity.^1^ Therapeutic approaches are exploring vagal stimulation as a way to rectify such cardiovascular conditions. However, the vagus nerve is approximately 80% sensory^2^ and contains motor efferent neurons projecting to various peripheral organs in addition to the heart.^3^^,^^4^^,^^5^ Cell bodies of cardiac vagal motor neurons projecting to the intrinsic cardiac nervous system (ICN) reside in two physiologically and anatomically distinct brainstem nuclei, the nucleus ambiguus (NA) and the dorsal motor nucleus of the vagus (DMV). While the DMV maintains ventricular contractility, the NA is primarily involved in regulating heart rate and rhythm.^6^^,^^7^^,^^8^ The DMV is required for the cardioprotective effect of remote ischemic preconditioning (RIPC).^6^ Identifying which molecular characteristics of the DMV are responsible for maintaining heart health and classifying their anatomical distribution provides precise therapeutic targets for selective vagal stimulation.

Stimulation of right-left and rostral-caudal subdivisions of the DMV has shown that the left DMV at the level of area postrema is responsible for changes in ventricular contractility.^9^ We sought to distinguish molecular properties of these cardiac-projecting DMV neurons at the transcriptional and protein level which may contribute to the DMV’s role in enhancing vagal tone and maintaining heart health. While neurons are classically characterized by their primary neurotransmitter released, neuromodulatory co-transmission is an emerging discipline. Our lab has discovered cholinergic neurons in the DMV that are simultaneously GABAergic^10^ and cholinergic neurons of the ICN that are simultaneously catecholaminergic.^11^ These findings suggest cardiac vagal motor neurons are transcriptionally primed to respond to environmental inputs, for example in response to RIPC.^10^ To identify ICN-projecting DMV neurons and characterize their molecular profiles anatomically we combined neural tracing with transcriptomic methods and validated our findings with immunofluorescence.

We determined the consistent transcriptional and protein character of ICN-projecting DMV neurons. Neuromodulators we assayed for include those known to play a role in cardiovascular regulation including glucagon-like peptide-1 (GLP-1),^6^ follistatin-1,^12^^,^^13^ PACAP,^14^ oxytocin,^15^ orexin,^16^ brain-derived neurotrophic factor (BDNF),^17^ bradykinin,^18^ nitric oxide,^18^ and CART.^19^^,^^20^ We expand upon previous findings highlighting the importance of anatomical subdivisions of DMV^9^ causing us to map the side and rostro-caudal level of cardiac-projecting DMV neurons. We investigated the transcriptomic patterns of the dorsal vagal complex (DVC), DMV, and NA, using multiple single cell scale approaches, including laser capture microdissection, tissue dissociation, qPCR, and RNAseq, as well as spatial transcriptomics. We sought to determine whether ICN-projecting DMV neurons participate in neuromodulatory co-expression at the transcriptional and protein levels, as well as to hypothesize which of these neuromodulators may be involved in maintaining heart health, and thereby be viable therapeutic targets.

## Results

### Spatial neuroanatomical analysis shows that the DVC contains unique transcriptomic profiles and markers relative to the rest of the brainstem

**Figure 1:**
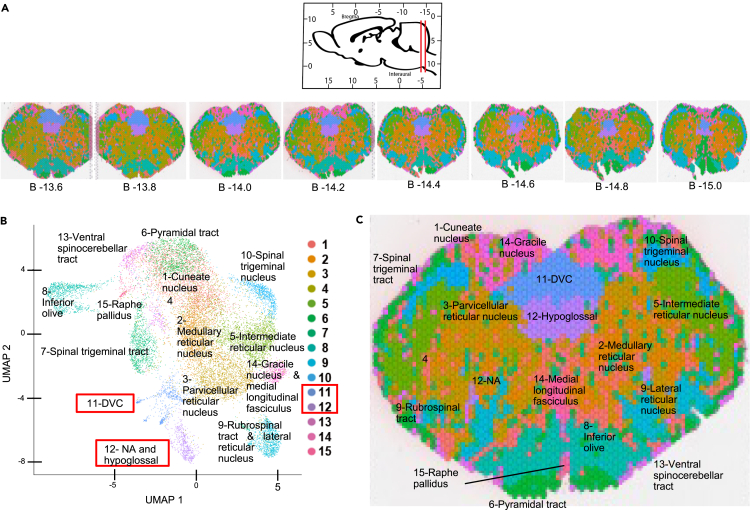
Spatial neuroanatomical analysis shows that the DVC contains unique transcriptomic profiles relative to the rest of the brainstem (A) Sagittal view of the rat brain with red lines indicating the most rostral (left) to the most caudal (right) sections collected to generate Visium spatial transcriptomics data. Visium sections with barcodes colored according to transcriptomic phenotype (see UMAP below) with respective mm distance from Bregma as indicated. (B) UMAP displaying Visium spatial transcriptomic phenotypes annotated according to the corresponding brainstem region. Transcriptomic phenotypes specific to the DVC and NA are outlined in red. (C) Transcriptomic phenotypes overlaid over one representative Visium section (B – 14.0) annotated according to brain region. DVC: dorsal vagal complex; NA: nucleus ambiguus. See also Table S1.

To identify whether the spatial transcriptomic profile of DMV is distinguishable from the larger transcriptomic landscape of the brainstem, we performed an unbiased clustering analysis of Visium spatial transcriptomics data to extract the transcriptomic profiles in our data indicative of distinct molecular phenotypes at intermediate levels of the medulla oblongata (Figure 1). Transcriptomic profiles were similarly detected on both sides of the brainstem, across eight tissue sections at different rostro-caudal levels (Figure 1A). The uniform manifold approximation and projection (UMAP) technique was used for dimensionality reduction and identification of clusters of transcriptomic profiles from the spatial transcriptomics data (Figure 1B). 15 transcriptomic profiles were found to correlate to 15 previously established neuroanatomical structures, indicating that the transcriptomic data reflect the distinct molecular characteristics and functions of these brainstem nuclei (Figure 1C). The transcriptomic profile anatomically specific to the broader brainstem region containing the DMV, the DVC, is cluster 11 (Figures 1B and 1C). The DVC has visceral sensory and autonomic motor nuclei of the Vagus nerve. The transcriptomic profile of the other cardiac vagal motor nucleus in the brainstem, the nucleus ambiguus (NA), is cluster 12 (Figures 1B and 1C). At this level of granularity (50 micron data points), the transcriptomic profile of another cholinergic nucleus of the brainstem, the hypoglossal nucleus, is also cluster 12 (Figures 1B and 1C). This analysis suggests that distinct molecular characteristics, detectable at the transcriptomic level, differentiate the DMV from the other two cholinergic nuclei of the brainstem, the nearby hypoglossal nucleus and the cardiac vagal motor neurons of the NA. These results also indicate that the spatial transcriptomic landscape of the brainstem is neuroanatomically specific and bilaterally symmetric. The molecular identities of these 15 clusters are presented in Table S1.

**Figure 2:**
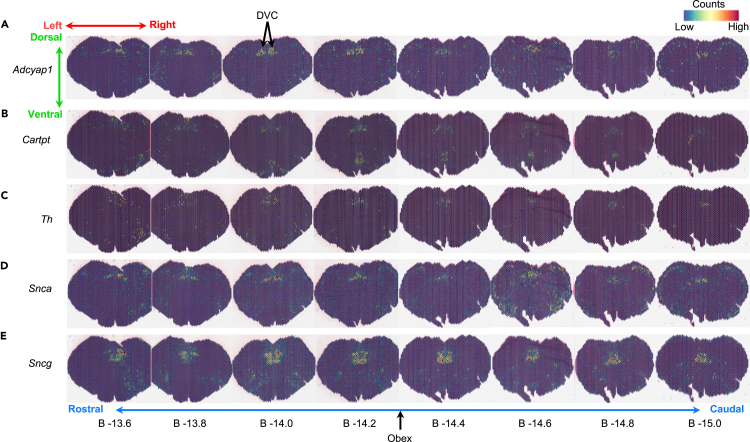
Spatial neuroanatomical analysis shows the DVC contains unique transcriptomic markers (A–E) Raw gene expression counts of (A) *Adcyap1*, (B) *Cartpt*, (C) *Th*, (D) *Snca*, and (E) *Sncg* shown in the context of the tissue to illustrate the specificity of these markers to the DVC relative to the rest of the brainstem.

The specific molecular markers distinguishing the region 11 containing DMV from the rest of the brainstem include *Igfbp5* (*p* = 3.71 x 10^−53^), *Sncg* (*p* = 7.71 x 10^−110^), *Snca* (*p* = 6.21 x 10^−157^), *Prph* (*p* = 7.56 x 10^−108^), *Zcchc12* (*p* = 1.41 x 10^−211^), *Atp1a1* (*p* = 6.27 x 10^−144^), *Adycap1* (*p* = 1.60 x 10^−264^), *Cartpt* (*p* = 7.8 x 10^−148^), and *Th* (*p* = 8.39 x 10-^275^) (Figure 2). These transcripts were distinguished by the highest average log2 fold change (1.44, 1.18, 1.17, 1.08, 1.08, 1.07, 1.01, 0.85, and 0.62, respectively). *Th* and *Sncg* are also detected in cluster 12. Otherwise, these transcriptomic markers are either lowly or not expressed in the barcodes associated with the NA or hypoglossal nucleus, suggesting these transcripts delineate molecular characteristics of DMV neurons from those of the other cholinergic nuclei of the brainstem (Figure 2). Expression of these transcripts is bilaterally symmetric but varies over the rostro-caudal extent of the DMV, with expression levels particularly high in the DMV region coextensive with the most caudal portion of area postrema (AP; bregma – 14.0).

### Single cell RNAseq analysis identified DVC-specific expression of transcription factors and inhibitors of NfkB signaling

**Figure 3:**
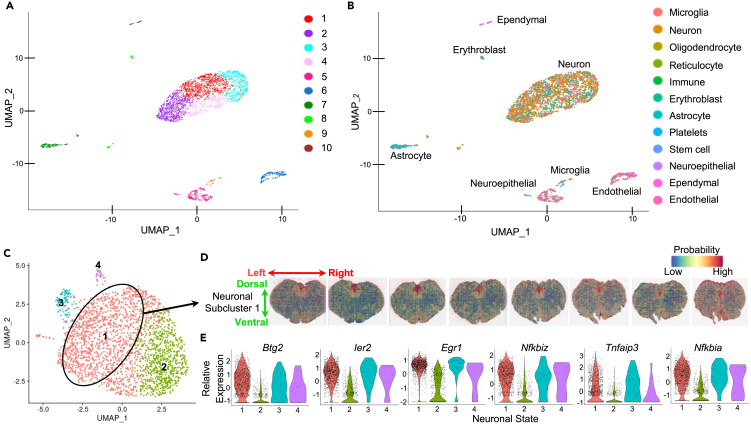
Single cell RNAseq identified DVC-specific expression of transcription factors and inhibitors of NfkB signaling (A and B) UMAP of sc-RNAseq data (A) annotated per transcriptomic cluster (1–10) and (B) annotated per cell type (according to canonical marker gene with maximum expression). (C) UMAP of sc-RNAseq data characterized as neurons from (B) annotated per neuronal subcluster (1–4). See also Figure S1 and Table S2. (D) Mapping of the sc-RNAseq data onto the spatial transcriptomics data for extracting tissue context. Color indicates probability of the transcriptomic profile of neuronal subcluster 1 from (C) expressed across the brainstem. (E) Relative expression levels of transcriptomic markers of DMV-specific neuronal subcluster 1 from (C).

We acquired sc-RNAseq data from the dorsal medulla region using 10x Genomics Chromium. UMAP (as aforementioned) was used to perform unbiased dimensionality reduction of the sc-RNAseq data of the dorsal medulla and to distinguish transcriptomic profiles within the data. This analysis yielded 10 transcriptomic clusters (Figure 3A). To determine which of these clusters is dominated by neuronal gene expression, expression levels of a variety of canonical cell type markers were examined (Figure S1A). This analysis resulted in classification of clusters 1–4 as primarily neuronal, cluster 5 as neuroepithelial, cluster 6 as endothelial, cluster 7 as astrocytic, cluster 8 as erythroblasts, cluster 9 as microglial, and cluster 10 as ependymal (Figure 3B). The data were subset for neuron-dominated clusters (1–4) and reanalyzed again using UMAP, which yielded 4 clusters (Figure 3C). The molecular identities of these 4 neuronal clusters are presented in Table S2. To identify which of these neuronal phenotypes from the dorsal brainstem is DVC-specific, we performed a transfer-based learning technique to identify anchors between our reference sc-RNAseq neuronal cluster data and our query spatial transcriptomics data. After integration with the Visium spatial transcriptomics data, the transcriptomic profile of neuronal cluster 1 from the sc-RNAseq data were predicted with high probability and anatomical specificity to be detected in the DVC region of the spatial transcriptomics data (Figure 3D). Neuronal cluster 2 from the sc-RNAseq data were predicted with high probability to be detected throughout the brainstem, however, not including the DVC region (Figure S1B). Instead, neuronal cluster 2 was predicted to have a very low probability of being present in the DVC region. Markers of neuronal cluster 1 include some transcription factors including *Btg2* (*p* = 3.27 x 10^−186^), *Ier2* (*p* = 1.83 x 10^−189^), and *Egr1* (*p* = 1.04 x 10^−186^), as well as some inhibitors of NfkB including *Nfkbiz* (*p* = 1.32 x 10^−147^), *Tnfaip3* (*p* = 1.26 x 10^−80^), and *Nfkbia* (*p* = 2.10 x 10^−154^) (Figure 3E)*.* This analysis indicated that, based on this differentiation, single cell samples specific to the DVC region could be identified from our sc-RNAseq data from dorsal brainstem tissue.

### Single cell gene expression analysis of laser capture microdissected brainstem neurons reveals that *Adcyap1* and *Cartpt* are selectively expressed by cardiac-projecting DMV neurons

We recognize that the DMV is a heterogeneous population with neurons projecting to various organs, serving the functions of multiple organs as well as acting as an integrative population for parasympathetic endocrine function.^21^ We investigated whether cardiac-projecting DMV neurons (identified by Fast Blue labeling from cardiac injections, as described in the STAR Methods) represent a distinct phenotype within the molecular landscape of DMV. We first isolated cardiac-projecting neurons using the retrograde Fast Blue tracer (described in the STAR Methods). In order to determine whether these neurons possess a unique molecular identity within the overall DMV population, we combined the single cell HT-RTqPCR and cardiac connectivity data in DMV cells. These results showed the cardiac-projecting cells to express several functionally significant transcripts predominantly and differentially.

**Figure 4:**
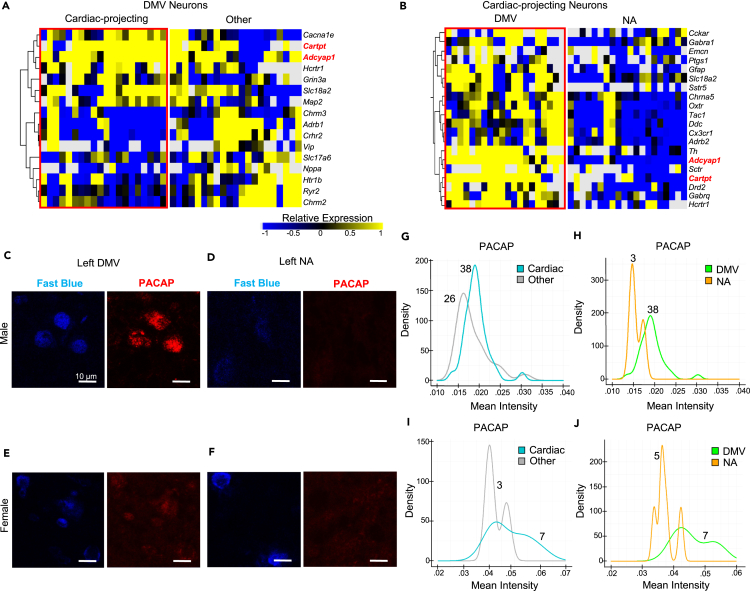
Single cell gene expression analysis of laser capture microdissected brainstem neurons reveals that *Adcyap1* and *Cartpt* are selectively expressed by cardiac-projecting DMV neurons (A and B) (A) Heatmap displaying relative expression of transcriptomic markers (ANOVA, *p* < 0.05) of (A) cardiac-projecting vs. other DMV neurons and of (B) DMV vs. NA neurons. (C–J) Confocal immunofluorescence images at 20X with 400% zoom of PACAP (right) expression in cardiac-projecting neurons of the (C and E) left DMV and (D and F) left NA at the level of area postrema in the (C and D) male and (E and F) female rat. Representative images are shown from *n* = 3 sections per sex. Kernel density estimates of the mean intensity levels of PACAP protein in (G and I) cardiac-projecting DMV vs. other DMV neurons and (H and J) cardiac-projecting DMV vs. NA neurons. Numbers indicate the number of PACAP-positive neurons in the corresponding region of a given section. See also Figures S2 and S3.

*Cartpt*, *Adcyap1*, and several other transcripts are significantly enriched (ANOVA *p* < 0.05; *p* = 0.018 and *p* = 0.011, respectively) in cardiac-projecting DMV neurons relative to other DMV neurons (Figure 4A). Cardiac-projecting neurons of the DMV are also significantly enriched for several transcripts relative to those of the NA, including catecholaminergic markers (*Slc18a2*, *Ddc*, and *Th*; *p* = 0.002, *p* = 9.01 x 10^−5^, and *p* = 9.32 x 10^−6^, respectively), and neuropeptides (*Cartpt* and *Adcyap1*; *p* = 8.42 x 10^−6^ and *p* = 1.47 x 10^−11^, respectively) (Figure 4B).

After identifying *Adcyap1*, *Cartpt*, and *Th* as being significantly enriched in cardiac-projecting neurons of the DMV relative to those of the NA, we sought to validate this finding at the protein level. We interrogated the relationship between protein expression levels of these neurotransmitter synthesis enzymes and neuropeptides in cardiac-projecting DMV neurons in both male and female. In both sexes, ChAT mean intensity values were positively correlated with those of PACAP and TH (Figure S2). We performed immunofluorescence staining of the DMV and the NA within the same tissue sections (Figures 4C–4F, S3A–S3D, and S3G–S3J). PACAP mean intensity is higher in cardiac-projecting DMV neurons as compared to non-cardiac-projecting DMV neurons as well as NA neurons in male and female (Figures 4G–4J). CART mean intensity was higher in cardiac-projecting DMV neurons as compared to non-cardiac-projecting DMV neurons (Figure S3E) as well as NA neurons (Figure S3F) in male and female (Figures S3K and S3L). In female mean intensity of CART was higher in cardiac-projecting NA neurons as compared to DMV neurons. CART protein and transcript (*Cartpt*) are molecular markers of cardiac-projecting neurons of the DMV relative to those of the NA in male, but not female. These findings further support that PACAP protein and transcript (*Adcyap1*) are molecular markers of cardiac-projecting neurons of the DMV, relative to other DMV neurons and relative to those of the NA in both sexes.

### Distinctive neuromodulatory co-expression distinguishes cardiac-projecting DMV neurons from other vagal motor neurons

**Figure 5:**
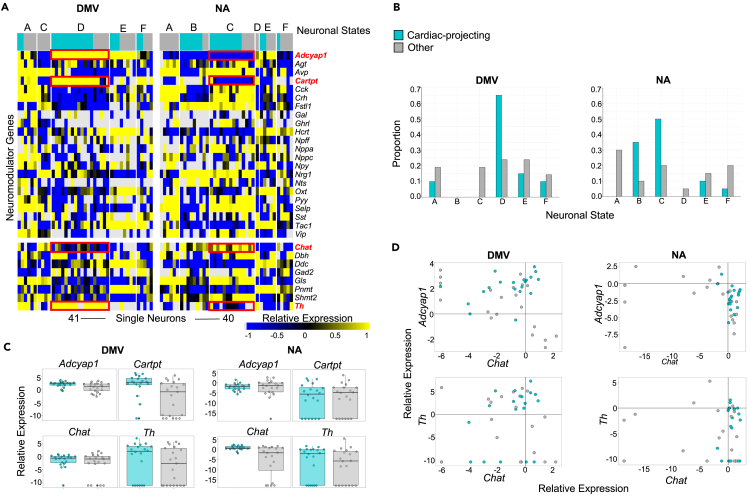
Distinctive neuromodulatory co-expression of cardiac-projecting DMV neurons relative to other vagal motor neurons (A) Normalized single neuron gene expression profiles of neuropeptides (top) and neurotransmitter synthesis enzymes (bottom) annotated by brain region (DMV/NA), connectivity to the heart (cardiac-projecting or other), and neuronal transcriptomic subtype (states A–F). Neuronal transcriptomic subtype D is most enriched for cardiac-projecting DMV neurons and expresses high levels of *Adcyap1*, *Cartpt*, and *Th*, but low levels of *Chat* (all boxed in red). See also Figures S4 and S5. (B) Proportion of cardiac-projecting and non-cardiac DMV (left) and NA (right) neurons corresponding to each neuronal transcriptomic subtype. (C) Relative gene expression levels of *Adcyap1, Cartpt, Chat,* and *Th* across cardiac-projecting vs. non-cardiac DMV (left) and NA (right) neurons. (D) Relative gene expression levels of *Adcyap1* vs. *Chat* (top) and *Th* vs. *Chat* (bottom) in cardiac-projecting vs. non-cardiac DMV (left) and NA (right) neurons.

To investigate neurotransmission from the DMV and NA to the ICN, we obtained HT-RTqPCR data across neuromodulatory genes, including neuropeptide precursors as well as neurotransmitter synthesis enzymes (Figure 5A). To identify transcriptomic phenotypes across DMV and NA in our LCM-qPCR data, hierarchical clustering was performed via Pearson correlation with complete linkage. Samples collected from both the DMV and the NA were present in nearly all of the clusters, with the exceptions being cluster B, which contained neurons exclusively from the NA, and cluster D, which contained neurons almost exclusively from the DMV (Figure 5B). The majority of cardiac-projecting DMV neurons in the LCM-qPCR data fall into cluster D, while the majority of cardiac-projecting NA neurons fall into cluster C. Of interest, DMV-specific cluster D is *Adcyap1+*, *Cartpt+*, and catecholaminergic (*Th+*) while NA-specific cluster C is *Adcyap1-*, *Cartpt-*, and cholinergic (*Chat+*) (Figure 5C). Similar to the protein expression data, cardiac-projecting DMV neurons show a positive correlation between *Chat* expression levels and both *Adcyap1* and *Th* expression levels (Figure 5D).

Of the remaining clusters, cluster A represents a transcriptomic phenotype which contains minimal cardiac-projecting DMV neurons, as well as non-cardiac-projecting neurons from both the DMV and NA. Cluster A is *Gls+* and co-expresses many neuropeptide precursor genes, except for *Hcrt* and *Nrg1.* Cluster B, containing predominantly cardiac-projecting neurons of the NA, is *Fstl1+*, *Nrg1+*, and *Chat+.* Clusters E and F represent two non-cholinergic transcriptomic phenotypes found in both the DMV and the NA, including cardiac-projecting and non-cardiac neurons. Cluster E is *Adcyap1+*, *Cartpt-*, *Hcrt+*, *Oxt+*, *Tac1+*, *Ddc+*, and *Pnmt+.* Cluster F is *Sst*+, *Ddc+*, *Gad2+*, and *Pnmt+.*

We compared the gene expression patterns of receptors as well as ion channels, neurotransmitter metabolism enzymes, transcription factors, and transporters between DMV and NA neurons (Figures S4 and S5). Our analysis indicates that DMV-specific cluster D and NA-specific cluster C express similar levels of GABAergic and glutamatergic receptor genes (Figure S4). NA-specific cluster C expresses higher levels of many different receptor genes, including those involved in signaling of angiotensin II, calcium, catecholamines, acetylcholine, glycine, neuropeptides, and androgen, as compared to DMV-specific cluster D. *Hcn3*, a pacemaker channel that mediates rhythmic firing in the DMV,^22^ is enriched in DMV-specific cluster D relative to cardiac-projecting neurons of cluster C in NA. There is no statistically significant difference in *Hcn3* expression between the cardiac-projecting neurons versus other neurons in DMV cluster D. Similarly, *Slc18a2* (VMAT2) expression is enriched in DMV-specific cluster D relative to clusters B and C. The choline transporter *Slc5a7* (CHT1) is expressed at relatively higher levels in clusters B and C (Figure S5).

### Cardiac-projecting PACAP+ neurons of the intermediate left DMV selectively express synuclein

**Figure 6:**
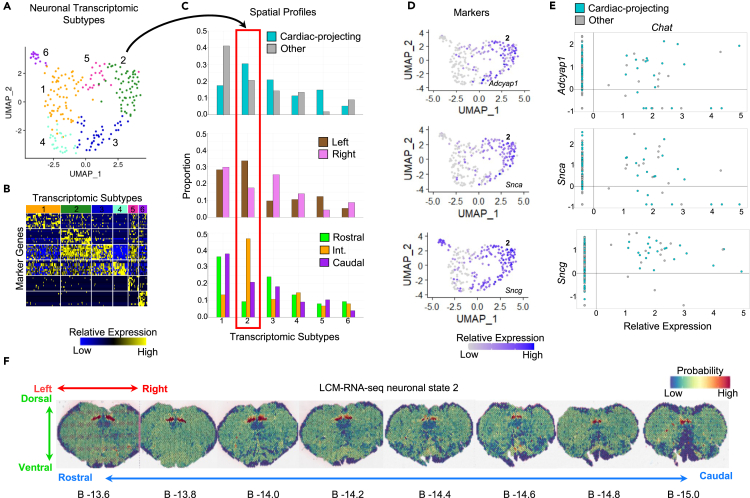
Cardiac-projecting PACAP+ neurons of the intermediate left lateral DMV selectively express synuclein (A) Distribution and clustering of neuronal transcriptomic phenotypes (top). (B) Normalized expression levels of top 10 marker genes per neuronal transcriptomic subtype (bottom). See also Figures S6 and S7, and Table S3. (C) Proportion of cardiac-projecting (top), left and right (middle), and caudal, intermediate (int.), and rostral (bottom) neurons corresponding to each transcriptomic subtype. Neuronal transcriptomic subtype 2 (boxed in red) shows the highest proportion of cardiac-projecting, left, and middle (rostro-caudally) DMV neurons. (D) UMAP colored for relative expression of subtype 2 markers *Adcyap1* (PACAP transcript; top), *Snca* (α-Synuclein; middle), and *Sncg* (gamma-Synuclein; bottom). (E) Relative gene expression levels of *Adcyap1* (top), *Snca* (middle), and *Sncg* (bottom) vs. *Chat* in cardiac-projecting vs. non-cardiac DMV neurons. (F) Mapping of the LCM-RNAseq data onto the spatial transcriptomics data for extracting tissue context. Color indicates probability of the transcriptomic profile of neuronal transcriptomic subtype 2 from (A and B) expressed across the brainstem.

To contextualize these findings within the broader landscape of DMV transcriptomic phenotypes, UMAP was used for unbiased dimensionality reduction and to identify neuronal phenotypes in the RNAseq data of microdissected DMV neurons. The results from this analysis showed that the single DMV neurons partition into six neuronal transcriptomic subtypes (Figure 6A). The molecular identities of these six neuronal transcriptomic subtypes are presented in Table S3. The top 10 marker genes for each neuronal transcriptomic subtype are distinct, indicating distinct neuronal transcriptomic subtypes (Figure 6B). The greatest proportion of cardiac-projecting DMV neurons belong to neuronal transcriptomic subtype 2 (Figure 6C, top). We also examined the distribution of left vs. right DMV neurons. The greatest proportion of left DMV neurons also belongs to neuronal transcriptomic subtype 2 (Figure 6C, middle). We determined whether there was a differential distribution of rostral, middle, and caudal DMV neurons within these neuronal transcriptomic subtypes (Figure 6C, bottom). Almost 50% neurons sampled from the intermediate region of the DMV were identified to belong to neuronal transcriptomic subtype 2. This distinct spatial profile of neuronal transcriptomic subtype 2 is consistent with previously reported descriptions of cardiac-relevant DMV neurons. To identify transcriptomic markers of neuronal transcriptomic subtype 2 as compared to the five other neuronal transcriptomic subtypes, we performed DEseq2 differential expression analysis. From this analysis, *Adcyap1* (*p* = 7.54 x 10^−16^), *Snca* (*p* = 1.08 x 10^−22^), and *Sncg* (*p* = 1.03 x 10^−17^) were determined to be transcriptomic markers of neuronal transcriptomic subtype 2 (Figure 6D). *Adcyap1*, *Snca*, and *Sncg* are found to be co-expressed with *Chat* in DMV neurons (Figure 6E). The expression levels of the top two markers for each neuronal transcriptomic subtype are shown overlaid over the UMAP in Figure S6. These results suggest *Adcyap1*, *Snca*, and *Sncg* are co-expressed with *Chat* in cardiac-projecting neurons of the DMV.

We sought to predict the neuroanatomical specificity of neuronal transcriptomic subtype 2 to the DMV region by performing anchor-based integration of our microdissected RNAseq cluster data with our spatial transcriptomics data, as described previously. Across all eight brainstem sections, the probability of detecting the transcriptomic profile of neuronal transcriptomic subtype 2 in the spatial transcriptomics data are high in the DMV region and relatively low elsewhere (Figure 6F). This analysis suggests that the transcriptomic profile of neuronal transcriptomic subtype 2 is predicted with high probability and high anatomical specificity to be delimited to the DMV region of the brainstem. We compared these neuronal transcriptomic subtypes to those previously described from single nucleus transcriptomics of DMV.^5^ Subtype 2 in our transcriptomic data correlates to the expression profile of Nppb and Trpv1 neurons from Tao et al. (Figure S7). Other DMV transcriptomic subtypes predominantly corresponding to cardiac-projecting neurons (subtypes 3 and 5) are most similar to Grp and Trpv1 neurons (Figure S7).

**Figure 7:**
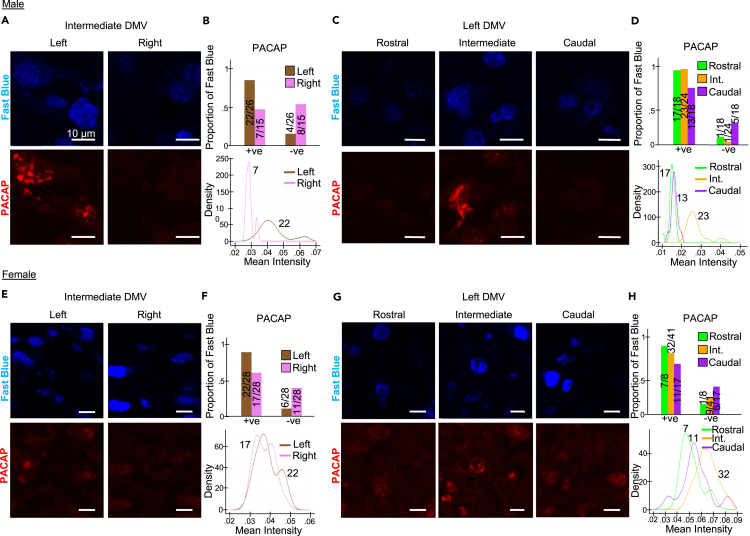
Cardiac-projecting neurons in the intermediate lateral DMV selectively express PACAP protein (A) Confocal immunofluorescence images at 20X with 400% zoom of PACAP protein expression in cardiac-projecting neurons of the left and right DMV at the level of area postrema in male rat. Representative images are shown from *n* = 3 sections. (B) Proportion of PACAP-positive and PACAP-negative cardiac-projecting neurons (top) and kernel density estimates of average intensity values of PACAP expression in cardiac-projecting neurons of the left and right DMV (bottom). Numbers indicate the number of PACAP-positive and PACAP-negative neurons in the corresponding region of a given section. (C) Confocal immunofluorescence images at 20X with 400% zoom of PACAP protein expression in cardiac-projecting neurons at the rostral, intermediate, and caudal levels of DMV in male rat. Representative images are shown from *n* = 3 sections. (D) Proportion of PACAP-positive and PACAP-negative Fast Blue neurons of the rostral, intermediate, and caudal DMV (top) and kernel density estimates of average intensity values of PACAP expression in cardiac-projecting neurons of the rostral, intermediate, and caudal left DMV (bottom). (E–H) Same as in (A–D) for female rat. See also Figures S8–S10.

We determined whether the spatial profile of the neuropeptide transcribed from *Adcyap1*, PACAP, as well as the protein transcribed from *Snca*, SNCA, is also anatomically specific to the cardiac-projecting DMV neurons at the protein level. To investigate this, we performed anti-PACAP and anti-SNCA immunofluorescence staining followed by confocal imaging. We detected PACAP protein expression in cardiac-projecting (Fast Blue) DMV neurons of the left and right DMV at intermediate rostral-caudal levels in both sexes (Figures 7 and S8). In male, the proportions of Fast Blue neurons positive for PACAP protein expression are higher in left versus right intermediate DMV and the mean intensity of PACAP protein expression in Fast Blue neurons is higher in intermediate left versus right DMV (Figure 7B, bottom). After identifying higher PACAP expression levels in left DMV, we performed a similar analysis across rostral, intermediate, and caudal levels of left DMV (Figure 7C). In male, the proportions of Fast Blue neurons positive for PACAP protein expression are comparable between rostral and intermediate levels, with a lower proportion of PACAP-positive cardiac-projecting neurons at more caudal levels, and a higher mean intensity level of PACAP protein expression in Fast Blue neurons within the intermediate region of DMV (Figure 7D). In female, the proportion of Fast Blue neurons positive for PACAP expression is higher in the left versus right intermediate DMV (Figures 7E and 7F, top). Mean intensity of PACAP protein expression in Fast Blue neurons did not differ much between left and right DMV in female (Figure 7F, bottom). Similar to our findings in male and in female proportions of Fast Blue neurons positive for PACAP protein expression are comparable between rostral and intermediate levels, with a lower proportion of PACAP-positive cardiac-projecting neurons at more caudal levels, and a higher mean intensity level of PACAP protein expression in Fast Blue neurons within the intermediate region of DMV (Figure 7H). We detected SNCA protein expression in cardiac-projecting Fast Blue DMV neurons of the left and right DMV at intermediate rostral-caudal levels in male (Figure S9). The proportions of Fast Blue neurons positive for SNCA protein expression are comparable between right and left intermediate DMV, with more SNCA positive neurons in the left DMV, and the mean intensity of SNCA protein expression in Fast Blue neurons is higher in intermediate left DMV of male (Figure S9D). After identifying higher SNCA expression levels in left DMV, we performed a similar analysis across rostral, intermediate, and caudal levels of left DMV (Figure S9F). The mean intensity level of SNCA protein expression in Fast Blue neurons is higher in the intermediate DMV with more SNCA positive Fast Blue neurons at intermediate levels in male. We performed a similar analysis in females (Figure S10). A higher proportion of SNCA-positive Fast Blue neurons was detected in caudal DMV and SNCA mean intensity values were higher in intermediate DMV in female.

Collectively, these analyses suggest that PACAP and *Adcyap1* are widely distributed molecular markers of cardiac vagal motor neurons of the DMV with particular association to the left intermediate DMV at both the transcriptional and protein levels.

## Discussion

We have discovered cardiac-projecting DMV neurons to express neuromodulatory transcripts, including *Adcyap1*, *Cartpt*, *Snca*, and *Sncg*, with potential to have significant effects at the heart. These factors distinguish cardiac-projecting DMV neurons from other DMV, NA, and surrounding brainstem neurons. Our results show that no single gene expression marker exclusively identifies cardiac-projecting DMV neurons distinguishing them from DMV neurons projecting to other peripheral organ targets. The neuroanatomical positioning of DMV cardiac-projecting neurons is widespread but concentrated at rostral-caudal levels coextensive with area postrema. Our findings rest on a combination of high throughput single cell and spatial transcriptomic results, with cardiac-specific single neuron PCR and RNAseq data, and we have confirmed these findings at the protein level with immunofluorescence staining. Transcriptomic markers of cardiac-projecting neurons of the DMV overlap across sexes and techniques, including *Adcyap1*, *Snca*, *Sncg*, and *Th*, which suggests mechanisms of vagal contributions to heart health may be similar across sexes. Some catecholaminergic markers, *Slc8a2* and *Dbh*, were only well detected in the male data. Confirmation of transcriptomic findings at the protein level for PACAP, TH, and ChAT co-localization suggests cardiac-projecting DMV neurons may be poised to alter their neuromodulatory profiles in response to environmental cues.

The secreted neuropeptide PACAP is likely a marker of cardiac health for several reasons. PACAP has been demonstrated to be detected in DMV neurons,^23^ to be protective against ischemia-reperfusion-induced apoptosis in cardiomyocytes,^14^^,^^24^^,^^25^ to be neuroprotective,^26^ to increase ICN neuron excitability,^27^^,^^28^ to potentiate cholinergic transmission in rat ICN neurons,^29^^,^^30^ and to co-localize with ChAT in axons innervating ICN neurons.^28^^,^^31^ Expression of the PACAP receptor, PAC1, has been detected in guinea pig^32^^,^^33^ as well as rat ICN neurons.^14^ While PACAP has been detected in DMV neurons and at the level of fibers in the ICN,^31^^,^^34^ thus far, PACAP expression in ICN-projecting DMV neurons is only a speculation made plausible by immunohistochemical detection of ChAT in PACAP-positive fibers of the guinea pig cardiac ganglia.^35^ To our knowledge, this is the first report of PACAP detection in cardiac-projecting DMV neurons.

The presence of transcripts for synucleins, catecholamines, and CART in cardiac vagal motor neurons of the DMV suggests the importance of these factors in synaptic transmission from the DMV to the heart. Global synuclein knockout has previously been shown to reduce activity of DMV neurons.^36^ Additionally, the enzymatic activity^37^ and expression levels^38^ of TH are increased in response to global synuclein knockout, suggesting a role for synucleins in regulating catecholaminergic transmission. CART has previously been highlighted as having a mitochondrial mechanism of neuroprotection^39^ and has been suggested to be implicated in the stress response^40^ as well as in cardiovascular regulation.^19^^,^^20^ CART has also been detected in caudal noradrenergic DMV neurons innervating the spleen.^41^ This work presents the first finding of *Adcyap1*, *Snca*, *Sncg*, *Cartpt*, and *Th* co-localization in cardiac-projecting DMV neurons.

The present results motivate extensive exploration of their specific implications within the immune landscape influenced by vagal cardiac innervation. We and others have suggested that the cardioprotective effects of Vagus nerve activity plausibly involve neuromodulatory influences on immune and inflammatory processes.^10^^,^^21^^,^^42^^,^^43^ The present findings provide several neuromodulatory candidates that may mediate these effects, including PACAP and CART, which showed significant enrichment in the DMV relative to the NA. Immune cells express receptors for PACAP and PACAP inhibits the production of pro-inflammatory cytokines and chemokines from macrophages, microglia, and dendritic cells.^44^ More specifically, PACAP inhibits the expression of two CXC chemokines (MIP-2 and IL-8) and four CC chemokines (MIP-1α, MIP-1B, MCP-1, and Rantes).^44^ PACAP also increases the transcription of anti-inflammatory IL-10 through cAMP-dependent CREB binding.^44^ The efferent arm of the cholinergic anti-inflammatory reflex (CAIP), originating from the DMV, canonically acts via the α7 nicotinic acetylcholine receptors (α7nAChR) expressed on immune cells and endothelial cells to inhibit NF-kB signaling, which we report evidence of in our data.^45^ PACAP potentiates α7nAChR currents and α7nAChRs are required for PACAP-induced NO production.^46^ However, CART is capable of suppressing inflammation even when nicotinic receptors are inhibited.^41^ Taken together, these findings suggest that PACAP may be involved in the α7nAChR-dependent CAIP, but that CART may act through a α7nAChR-independent mechanism to elicit an anti-inflammatory effect. Our results suggest that the enrichment of PACAP and CART in the cardiac-projecting DMV neurons suggests potential involvement of these neuromodulators in facilitating immunomodulation at the level of the heart under pathological conditions, for example in heart failure. TNF-α, IL-1β, and Il-6 have all been suggested to lead to myocardial depression by acting directly on myocytes, and TNF-α can produce immediate and delayed negative inotropic effects on myocardial tissue leading to left ventricular dysfunction.^43^ Vagus nerve stimulation significantly inhibits the increased expression levels of TNF-α, IL-6, HMGB-1, and IL-17A in post-ischemic myocardial tissue, increases anti-inflammatory IL-10 levels in both the ischemic and nonischemic regions, reduces the number of infiltrated macrophages, and decreases the signal intensity for cytokines involved in the recruitment of neutrophils (LIX) and macrophages (MCP-1) in the heart in rat models of ischemia/reperfusion injury.^47^^,^^48^^,^^49^ Considering that DMV stimulation alone ameliorates cardiac pathology and improves function post-ischemia,^6^^,^^50^ our results suggest a potential role for PACAP and CART in at least partly contributing to DMV-mediated cardioprotection through modulation of the immune microenvironment post-cardiac injury.

In agreement with our recently published work,^10^ we show that DMV neurons cannot be characterized by only one neuromodulator and that this principle applies to cardiac-projecting DMV neurons. When we consider a combination of transcripts (*Adcyap1*, *Cartpt*, *Th*, *Snca*, and *Sncg*) we can enrich for a subset of cardiac-projecting neurons more selectively than using any individual marker alone. Our results suggest a combinatorial neuromodulator heterogeneity even within the cardiac-projecting DMV neurons, pointing to challenges in defining cardiac-projecting neurons as a uniform molecularly defined subtype. Similar to Nissl and HRP geographic differences in morphological descriptions of DMV neurons, we observed regional differences in the distributions of neuronal phenotypes throughout the DMV at the transcriptomic and protein levels. Descriptions of morphological differences in the cells of the rostral, middle, and caudal subdivisions of the DMV^51^ thus may correlate with molecular differences. For example, Fox and Powley noted a distinct increase in soma area at the middle rostral-caudal level of DMV, where we define a distinct neuronal molecular phenotype. Quantitatively, cardiac-projecting neurons of the DMV span >2 mm caudal to the rostral pole of AP (rAP) and only <500 μm rostral to rAP. PRV reports have previously alluded to the bias within the DMV for cardiac neurons to be enriched caudal to obex.^52^

Future work is needed to resolve the significance of the catecholamine-synthetic enzyme *Th/*TH and its transporter *Slc8a2* in cardiac-projecting DMV neurons. This work also inspires further investigation of the role of *Adcyap1*, *Cartpt*, *Snca*, *Sncg*, and *Th* in heart failure and in cardioprotective physiological maneuvers like RIPC. As recent work has shown immediate and sustained increases in vagal preganglionic neuron activity during and after exercise,^53^ opportunities lie in investigating the functional role of these neuromodulators in exercise at both the DMV neuronal level and at their targets at the heart. Increased cardiac vagal activity during exercise maintains cardiac function via neuromodulators other than acetylcholine, as atropine does not affect any cardiac parameters during exercise, but VIP antagonism significantly reduces coronary artery blood flow to a similar level to vagal denervation.^54^ Similar studies could be performed with PACAP antagonism to gain an understanding of its functional significance. It seems likely that the nuances of maintaining heart health may be slightly altered across sexes and species, but the main mechanisms are intact. Since the cardiac-projecting DMV neurons are transcriptionally distinct from the rest of the brainstem, the specific transcriptomic profiles presented here can be mined from whole brain sc-RNAseq datasets or deconvoluted from bulk transcriptomic datasets to identify and learn more about the neurons of the DMV, and to infer mechanisms involved in their maintenance of heart health.

### Limitations of the study

Our study focused on retrograde labeling of central preganglionic DMV neurons projecting to the heart. Follow-on studies can build on our results by taking an anterograde labeling approach to characterize the post-ganglionic targets of cardiac-projecting DMV neuronal subtypes characterized herein. A previous study pursued such an approach for isolating gut-projecting vagal preganglionics using retrograde labeling to identify specific markers, followed by anterograde tracing to verify their peripheral organ targeting.^5^ Our study was performed on rats. Inter-species differences in DMV transcriptomic profiles remain to be determined. A study of 79 microdissected human brain regions showed that PACAP was detectable in all brain nuclei investigated, with the highest concentrations in the DVC.^55^ This finding is consistent with our results as well as Tao and colleagues that detected PACAP expression in most gut-projecting DMV clusters in mouse.^5^ A previous study used fluorescence *in situ* hybridization to show that 96% of mouse NA neurons lack *Adcyap1* gene expression.^56^ In addition, the single cell transcriptomic analysis of mouse NA neurons showed *Vip*, *Nts*, and *Crhr2* to be markers of neuronal subtypes in NA. Our findings of rat NA gene expression patterns are consistent with these results. Our gene and protein expression results show that, in the rat brainstem, PACAP is significantly enriched in the cardiac-projecting DMV neurons, whereas *Vip*, *Nts*, and *Crhr2* gene expression is relatively lower in cardiac-projecting DMV versus NA neurons.

Within the broader context of vagally mediated autonomic regulation, there is emerging data on species differences in the morphological and functional aspects of the vagal postganglionic neurons of the heart.^57^ Vagal preganglionics in the DMV are likely to show molecular and functional differences across species as well. Our findings are derived from combining results across different single cell transcriptomic technologies including single cell dissociation and spatial approaches. An examination of gene expression patterns at a protein level, particularly in mapping the somatic versus synaptic differences in protein expression, can further illuminate the circuit-level functions of cardiac-projecting DMV neurons.

## STAR★Methods

### Key resources table

**Table:** 

REAGENT or RESOURCE	SOURCE	IDENTIFIER
**Antibodies**		

Mouse anti-ChAT	NovusBio	NB110-89724; RRID: AB_1216476
Chicken ant-TH	Abcam	Ab134461
Mouse anti-PACAP	Santa Cruz	Sc-166180; RRID: AB_2289234
Goat anti-CART	FisherSci	AF163-SP
Chicken anti-SNCA	NovusBio	NBP2-25146SS

**Chemicals, peptides, and recombinant proteins**		

5% retrograde Fast Blue tracer	Polysciences	17740–1
SuperScript VILO Master Mix	Thermo Fisher Scientific	11755050
Taqman PreAmp Master Mix	Thermo Fisher Scientific	4391128

**Critical commercial assays**		

Adult Brain Dissociation Kit, mouse and rat	Miltyni	130-107-677
Chromium Next GEM Single Cell 3′ Kit v3.1	10x Genomics	1000269
Visium Spatial Gene Expression Slide & Reagents Kit	10x Genomics	1000187

**Deposited data**		

Spatial RNA-seq	GEO	GSE260847
Single cell RNA-seq	GEO	GSE260849
LCM-qPCR	GEO	GSE260845
LCM-RNA-seq	GEO	GSE260846

**Experimental models: Organisms/strains**		

Model organism: Sprague-Dawley rat	Envigo	N/A

**Software and algorithms**		

Real-Time PCR Analysis Software	Fluidigm	https://www.fluidigm.com/software
Seurat R package	Bioconductor Packages	https://satijalab.org/seurat/
R version 4.3.1	The R Foundation for Statistical Computing	https://www.r-project.org/

### Resource availability

#### Lead contact

Further information and requests for resources and reagents should be directed to and will be fulfilled by the lead contact, Rajanikanth Vadigepalli (rajanikanth.vadigepalli@jefferson.edu).

#### Materials availability

This study did not generate new unique reagents.

#### Data and code availability

• LCM-qPCR, LCM-RNAseq, spatial RNAseq, and single cell RNAseq data have been deposited at the Gene Expression Omnibus (GEO) database and are publicly available as of the date of publication under accession numbers GSE260845, GSE260846, GSE260847, and GSE260849, respectively.
• Original code has been deposited at Github at: https://github.com/Daniel-Baugh-Institute/DMV-Neuromodulator-Expression.
• Any additional information required to reanalyze the data reported in this paper is available from the lead contact upon request.

### Experimental model and study participant details

12-week-old male and female Sprague-Dawley rats (Envigo) were used for these experiments. All animals were housed in pairs. Animal facilities were maintained in a temperature and humidity-controlled room with 12/12 h light cycles (lights on at zeitgeber time = 0). Experimental procedures were carried out one week following animal arrival at our facility. All work was performed in agreement with protocols accepted by the Thomas Jefferson University Institutional Animal Care and Use Committee.

### Method details

#### Dorsal brainstem tissue dissociation and 10x genomics sc-RNAseq

We generated a single cell suspension from the dorsal medulla including the DMV by a dissociation protocol for adult rat brain,^58^ and viability was determined by calcein AM stain. The 4 samples included pools of 4 left and right dorsal brainstem from 12 week old male Sprague-Dawley rats. We obtained sc-RNAseq data using 10x Genomics Chromium assay, available as a TJU core facility service.

#### Spatial RNAseq of brainstem using 10x genomics Visium

Adult male Sprague-Dawley rat brainstem 10 μm sections evenly spaced 200 μm apart were put on 2 Visium slides (8 sections total). Permeabilization, barcoding, library construction, and sequencing were performed for each 50 μm barcoded spot. We did this throughout the extent of the brainstem including the DMV at the level of Area Postrema.

#### Fast Blue retrograde tracing and laser capture microdissection

To identify the transcriptional phenotypes and molecular characteristics distinguishing cardiac-projecting DMV neurons from other DMV neurons as well as cardiac vagal motor neurons of the NA, 5% retrograde Fast Blue tracer (FB) was injected into the ICN of 10 week old Sprague-Dawley rats. The rats were anesthetized with Isoflurane, their tracheas were intubated, and the rats were ventilated artificially with room air supplemented with oxygen. The chest was opened through the 2nd to 4th intercostal space. The ICN on the left atrium, near the SAN and hilum^59^ was injected with FB (25–30 μL/animal total in up to 4 sites). Two weeks later the animals were sacrificed by Isoflurane, and the brains were embedded in optimal cutting temperature (OCT) and frozen. Each brain was cryosectioned at 20 μm, placed on glass slides, stained with 0.0001% Cresyl Violet. Fast Blue-positive (cardiac-projecting) and Fast Blue-negative (non-cardiac-projecting) neurons from the DMV and the NA were identified under fluorescence (365 nm excitation and 420 nm emission) and acquired by Laser Capture Microdissection (LCM).^10^^,^^11^^,^^60^ Our selection criteria for LCM of single Fast Blue neurons included the high intensity and uniform distribution of Fast Blue labeling in the cytoplasm visible under the UV filter. This selection criteria was confirmed to accurately cluster unique molecular phenotypes distinguishing cardiac-projecting neurons from the rest of the DMV as well as from the NA. Samples were collected and stored on LCM caps at −80 until lysed with appropriate lysis reagents depending on downstream assay (RNAseq or HT-RTqPCR).

Our use of Fast Blue labeling by injection into the ICN region in rat greatly benefited by our recent studies of the rat ICN. We developed the first comprehensive 3D anatomical, molecular, and functional maps of the ICN in rat and pig hearts using tract tracing, single cell RNAseq, high-dimensional imaging, and 3D structural model reconstruction.^11^^,^^61^^,^^62^ These results provide clear anatomical landmarks for the present injections. In previous studies we identified Fast Blue as an ideal tracer to identify the vagal neurons innervating the heart.^11^^,^^62^ We previously optimized the tracer injection (concentration, volume, and precision) to prevent labeling by potential leakage from the cardiac sites of injection across multiple animals.^63^^,^^64^^,^^65^^,^^66^

#### Sc-RNAseq of LCM-acquired single DMV neurons (LCM-RNAseq)

239 single neurons positively and negatively labeled for Fast Blue retrograde tracer were obtained from the DMV via LCM. These samples were used to generate sc-RNAseq (LCM-RNAseq) data of 32,883 genes using a protocol we developed specifically for laser capture microdissection.^11^^,^^67^ Microdissected cells were processed for reverse transcription which produces the first and second cDNA strands in a single incubation, and this ds-cDNA has partial sequencing adapters at both ends. For library preparation, we followed the steps recommended in the research protocol (https://doi.org/10.21203/rs.3.pex-962/v1) for fresh frozen tissue with slight modifications, including 10 μL instead of 5 μL of lysis buffer and 22 cycles instead of 18 cycles of PCR amplification in step 3.

Fastq files were produced from raw sequencing data or base call files (BCLs) using the bcl2fastq program from Illumina. The polyA tail and G overhang were trimmed and the UMIs were extracted. Genome sequence indexing and alignment of reads to the Ratticus Norvegicus reference genome were performed using STAR software.^68^ The Subread R package^69^ feature count algorithm (featureCounts) was used to generate the count matrix of reads to genomic features, including genes and exons. Single neuron samples had an average read depth of 842,061 reads/sample, an average UMI count of 9,166 counts/sample, and a median of 2,175 detectable genes/sample. See “quantification and statistical analysis” section for data filtration details. The final count matrix consisted of 227 samples and 14,066 genes.

#### HT-RTqPCR of LCM-acquired single DMV and NA neurons (LCM-qPCR)

96 single neurons positively and negatively labeled for Fast Blue retrograde tracer were obtained from the DMV and NA via LCM. These samples were used to generate RT-qPCR (LCM-qPCR) data of 192 genes. For single cell high-throughput real-time LCM-qPCR we used the Fluidigm Biomark system that automates qPCR reactions in nanoliter volumes, reducing the amounts of sample and reagent required, which is compatible with TaqMan and EvaGreen chemistry. We have extensive experience with this and have used it in the brain and ICN previously (e.g.,^11^^,^^60^^,^^61^^,^^70^).We optimized this approach to achieve sensitivity and cell type specificity.^60^

SuperScript VILO Master Mix (Thermo Fisher Scientific, Waltham, MA) was used for reverse transcription of single neurons in lysis buffer. Amplification of genes was performed for 22 cycles using TaqMan PreAmp Master Mix as per the manufacturer’s protocol (Applied Biosystems, Foster City, CA, USA). Amplicons were detected by using Evagreen intercalated dye. BLAST^71^ was used to design intron-spanning PCR primers when appropriate. Primers were designed based on select genes involved in signal transduction (neurotransmitter synthesis enzymes, neuropeptide precursors, receptor subunits, ion channels, and transporters) and cell type identification. PCR was performed using 96.96 BioMark Dynamic Arrays (Fluidigm, South San Francisco, CA, USA) which allows for identical reaction conditions during measurement of multiple mRNAs and samples simultaneously. Each PCR run was amplified 30 times (15 s at 95°C, 5 s at 70°C, 60 s at 60°C). Real-Time PCR Analysis Software (Fluidigm) was used to calculate Ct values. Two 96 x 96 BioMark Arrays were used to measure gene expression across all 192 genes and 96 samples throughout the DMV and the NA, with half cardiac-projecting and half not cardiac-projecting. Each chip contained the same serial dilution gene set to verify reproducibility and test for technical variability.

Melt-curve analysis was used to examine the quality of the PCR results. Samples and genes with more than 30% failed reactions were filtered out. After data filtration, 81 single neuron samples (41 cardiac projecting) and 173 genes remained. Within each sample, DCt values were obtained by normalization of Ct values against a median expression level of a subset of robustly expressed genes with greater than 75% working reactions. Median sample expression values were chosen over reference genes after analysis of housekeeping genes as compared to median sample expression values using the selectHKs function in the NormqPCR package in RStudio. Within each gene, DCt values were median centered as well to obtain DDCt values. DDCt values were multiplied by negative 1 to convert these changes in Ct to expression level.

The following equation was used to calculate -DCt values for each gene:

−DCtgene = (median sample expression) − Ctgene.

The -DCt data were then rescaled using the median across all samples within a gene using the following equation:

−DDCtgene = −(DCtsample−DCtacross−sample−median).

#### Integration of spatial RNAseq and sc-RNAseq datasets

For the brainstem Visium data, anchor-based integration was performed using Seurat commands, which enabled the probabilistic transfer of transcriptional phenotype annotations from the 10x sc-RNAseq and LCM-RNAseq datasets to the 10x Visium dataset, using *FindTransferAnchors* and *TransferData* functions. The *SpatialFeaturePlot* function was used to visualize the probabilistic enrichment of each transcriptional phenotype annotation.

#### Heatmaps and scatterplots

For heatmaps and scatterplots of relative gene expression, normalized data were plotted using *myHeatmapByAnnotation* and *scatterGenes* functions, respectively, in the DataVisEasy R package.

#### Comparison of DMV neuronal transcriptomic subtypes to those of Tao et al. 2021

Metaneighbor analysis workflow^72^ was used to compare average normalized gene expression of each DMV neuron cluster from Table S1 of Tao et al. study^5^ to our normalized LCM-RNAseq gene expression matrix annotated for cardiac-projecting DMV neuronal transcriptomic subtypes. The *variableGenes* function was used to determine the top quartile (543 genes) of the most variable genes compared to their median expression and the *MetaNeighborUS* function was used to perform the similarity analysis and generate the cell type-by-cell type mean AUROC matrix comparing the DMV transcriptomic subtypes from our study to those of Tao et al. 2021.

#### Immunofluorescence

20 μm sections of frozen brainstem and were mounted on glass slides. DMV regions were fixed in 4% paraformaldehyde for 15 min and rinsed in PBS for 5 min three times. Sections were permeabilized for 15 min using 0.2% Triton X-100 and rinsed in PBS for 5 min two times. Blocking was done for 1 h in 10% normal goat serum at room temperature, followed by one 5-min PBS wash. Primary antibody incubation occurred overnight at 4°C. Primary antibodies included anti-ChAT (NB110-89724; 1:1000), anti-TH (ab134461; 1:500), anti-PACAP (sc-166180; 1:500), anti-CART (AF163-SP), and anti-SNCA (NBP2-25146SS). Sections were washed three times with PBS for 5 min each and then incubated with secondary antibody for 1 h and 45 min at room temperature, followed by three more washes for 5 min each. ProLong Diamond Antifade was used to coverslip and the slides were cured at room temperature overnight in the dark.

#### Confocal imaging

A Zeiss LSM 780 mounted on a Zeiss Axio Observer inverted microscope was used to generate confocal images. To determine the image acquisition parameters and to capture the images, the Zeiss ZEN 2011 software package associated with the LSM 780 was used. Lasers 405 nm (Fast Blue), 488 nm, 555 nm, and 647 nm were used for image acquisition. The range of approved signal levels were set prior to image acquisition using the range indicator function built into the Zeiss software. Images were acquired at a pixel resolution of 1,024 × 1,024 at 8-bit color depth with a line scan and averaging intensities from four scans of the same area.

### Quantification and statistical analysis

For statistical analyses of the LCM-qPCR data, a two-factor ANOVA followed by Tukey HSD was performed to compare cardiac-projecting neurons in the DMV only to non-cardiac-projecting neurons of the DMV, and those of the DMV to those of the NA. For hierarchical clustering of LCM-qPCR data, Pearson’s complete linkage was used to cluster all of the data in an unbiased manner. RStudio was used for all statistical analyses and data visualization.

#### Data processing with Seurat package

We used the Seurat package in R to identify transcriptional phenotypes in the LCM-RNAseq and 10x sc-RNAseq data. This approach involves filtration, normalization, dimensionality reduction, clustering of phenotypes, marker gene detection, and statistical analyses using the Seurat toolkit in R.^73^ For the LCM-RNAseq and 10x Genomics sc-RNAseq datasets, genes that were present in less than 3 cells were filtered out. Cells that expressed less than 200 genes or more than 10 percent mitochondrial genes were filtered out. After filtering, 227 cells throughout the DMV, including half cardiac-projecting and half not cardiac-projecting, passed quality control and were used in analysis of LCM-RNAseq data across 14,066 genes. For the 10x Genomics sc-RNAseq data, samples were further filtered to remove samples with both low quality degraded cells as well as potential doublets. Cutoffs for counts and features were determined by violin plots showing cell density as a function counts and features. In the male 10x sc-RNAseq data, a cutoff of 1,000–30,000 counts and 3,000–5,000 genes was applied. After filtering, data on a total of 18,133 genes and 3,195 samples remained, with an average of 340,895 reads/sample, an average of 7,706 UMI counts/sample, and a median of 1,780 detectable genes/sample. For both LCM-RNAseq and 10x Genomics sc-RNAseq datasets, UMI counts were normalized to total UMI counts (library size), multiplied by a factor of 10,000, and log transformed using the *NormalizeData* function. Using the *ScaleData* function, this normalized data were then z-scored. The *FindVariableFeatures* function was used to identify the top 2,000 highly variable genes and these selected genes were used to calculate principal components using the *RunPCA* function. The *ElbowPlot* function was used to identify the top principal components and these were selected for dimensionality reduction, clustering, and visualization with UMAP. Transcriptional phenotypes were identified by clusters having similar gene expression profiles and these clusters were detected by the *FindClusters* function, which uses the Louvain method for community detection. Differentially expressed genes were discovered by the Wilcoxon rank-sum test and DEseq2, both implemented by the *FindMarkers* function.

For the 10x Genomics Visiums spatial RNAseq data, the Seurat package in R was also used for normalization, clustering, dimensionality reduction, visualization, and integration. Normalization was performed using the *sctransform* function,^74^ which builds regularized negative binomial models of gene expression to account for technical noise while preserving biological variance. *RunPCA, FindNeighbors, FindClusters, RunUMAP, DimPlot* and *SpatialDimPlot* functions were used to run PCA, cluster, reduce dimensionality, and visualize clusters. The *SpatialFeaturePlot* function was used to visualize expression of specific genes in a spatial context.

## Supplemental information

Document S1. Figures S1–S10

Table S1. Spatial transcriptomic profiles of the brainstem, related to Figure 1

Table S2. Molecular identity of neuron-dominated transcriptomic clusters of single cells dissociated from the dorsal brainstem, related to Figure 3

Table S3. Transcriptomic phenotypes of laser capture microdissected cardiac-projecting neurons of the DMV, related to Figure 6
