# Supplementary material for: Neuromodulatory co-expression in cardiac vagal motor neurons of the dorsal motor nucleus of the vagus
Source: iScience. 2024 Jul 19;27(8):110549. doi: 10.1016/j.isci.2024.110549 (PMC11338141; doi:10.1016/j.isci.2024.110549)
Supplement: Document S1. Figures S1–S10 [file mmc1.pdf]

iScience, Volume *27*

Supplemental information

Neuromodulatory co-expression

in cardiac vagal motor neurons

# of the dorsal motor nucleus of the vagus

Eden Hornung, Shaina Robbins, Ankita Srivastava, Sirisha Achanta, Jin Chen, Zixi Jack Cheng, James Schwaber, and Rajanikanth Vadigepalli

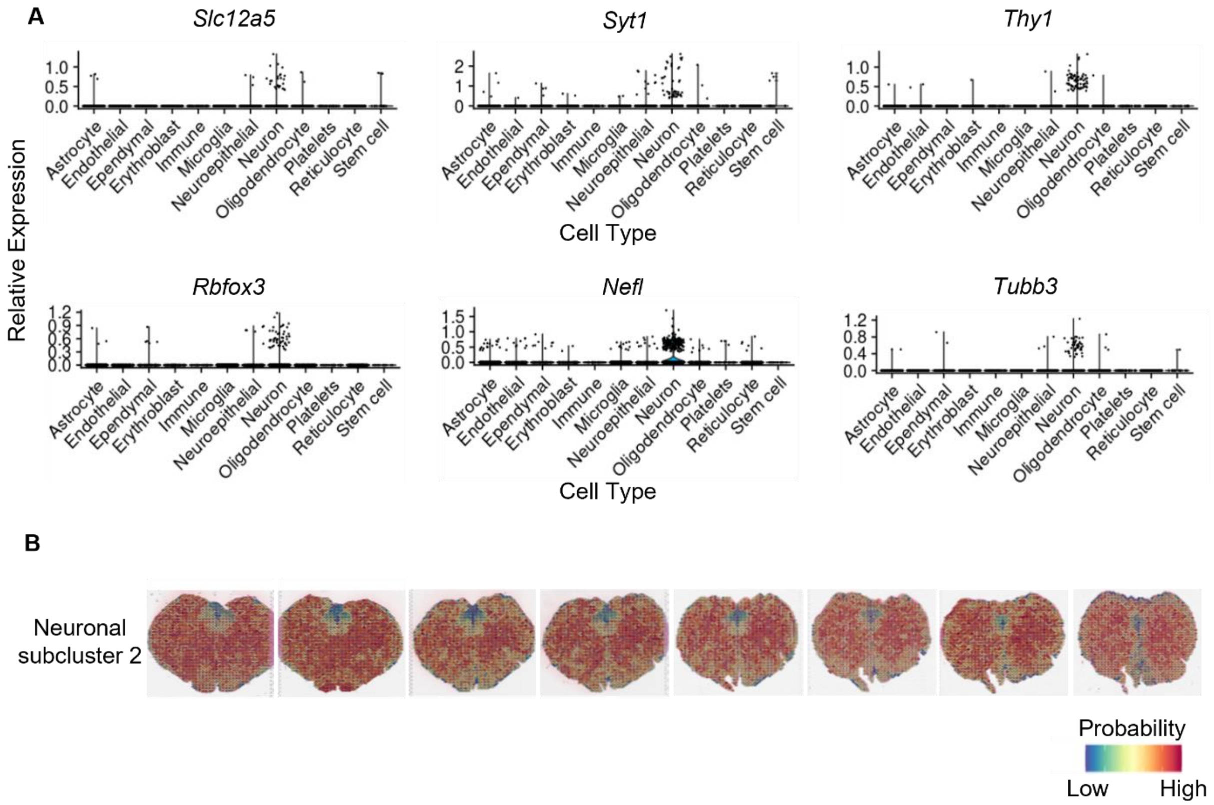

Figure S1. Detection of neuronal markers in single cell RNAseq data from dissociated dorsal brainstem tissue encompassing DMV, related to Figure 3. (A) Relative expression of neuronal marker genes *Slc12a5, Syt1, Thy1, Rbfox3, Nefl,* and *Tubb3* grouped per cell type annotation assigned according to maximum marker gene expression level. (B) Mapping of the sc-RNAseq data onto the spatial transcriptomics data for extracting tissue context. Color indicates probability of the transcriptomic profile of neuronal subcluster 2 from (Figure 3C) expressed across the brainstem.

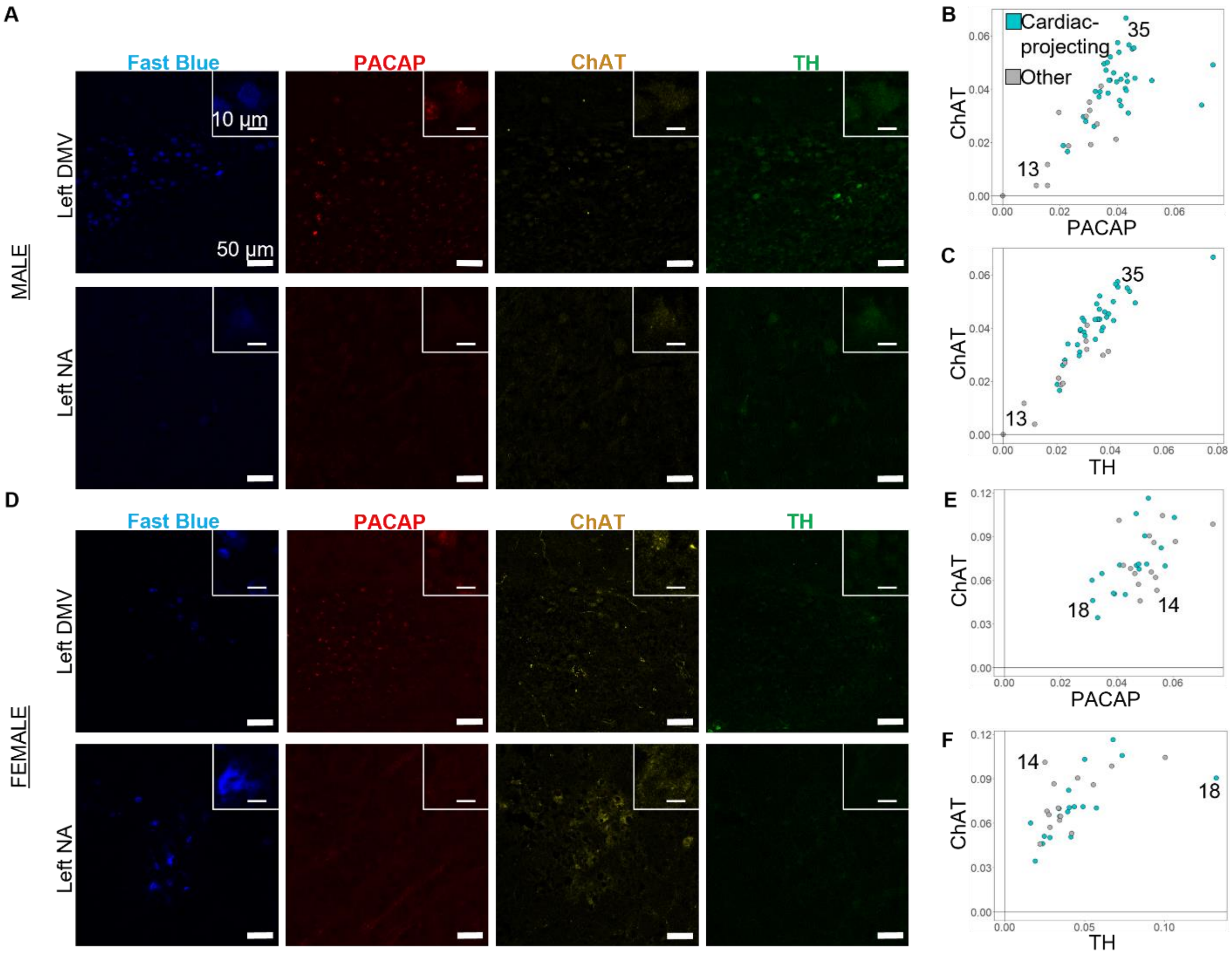

Figure S2. PACAP and TH are positively correlated with ChAT in cardiac-projecting DMV neurons, related to Figure 4. (A) Confocal immunofluorescence images at 20X of PACAP, ChAT, and TH protein expression levels in left DMV (top) and left NA (bottom) at the level of Area Postrema in male rat. Zoomed in insets are at 400 % zoom. Representative images are shown from n = 3 sections. (B-C) Mean intensity values of (B) ChAT vs. PACAP and (C) ChAT vs. TH across cardiac-projecting and other DMV neurons as shown in A. (D-F) Same as in (A-C) for female rat.

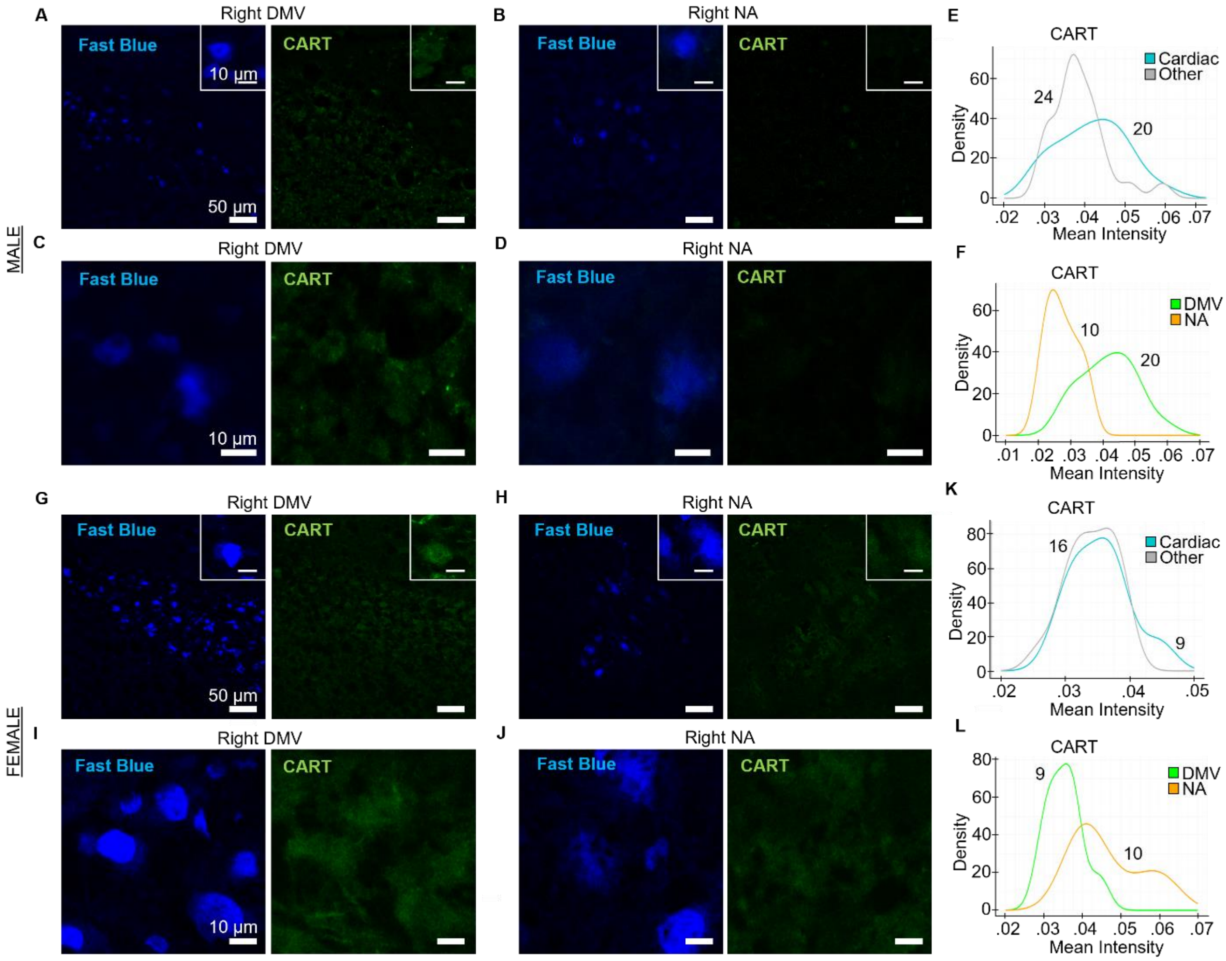

Figure S3. CART is selectively expressed by cardiac-projecting DMV neurons in male but not female rat, related to Figure 4. Confocal images at 20X of CART protein expression in tissue sections of (A) right DMV and (B) right NA at the level of Area Postrema in male rat. Representative images are shown from n = 3 sections. (C-D) Confocal images as in A-B at 400 % zoom. Kernel density estimates of mean intensity levels of CART protein expression in (E) cardiac-projecting DMV vs. other DMV neurons and (F) cardiac-projecting DMV vs. NA neurons. Numbers indicate the number of CART-positive neurons in the corresponding region of a given section. (G-L) Same as in A-F for female rat.

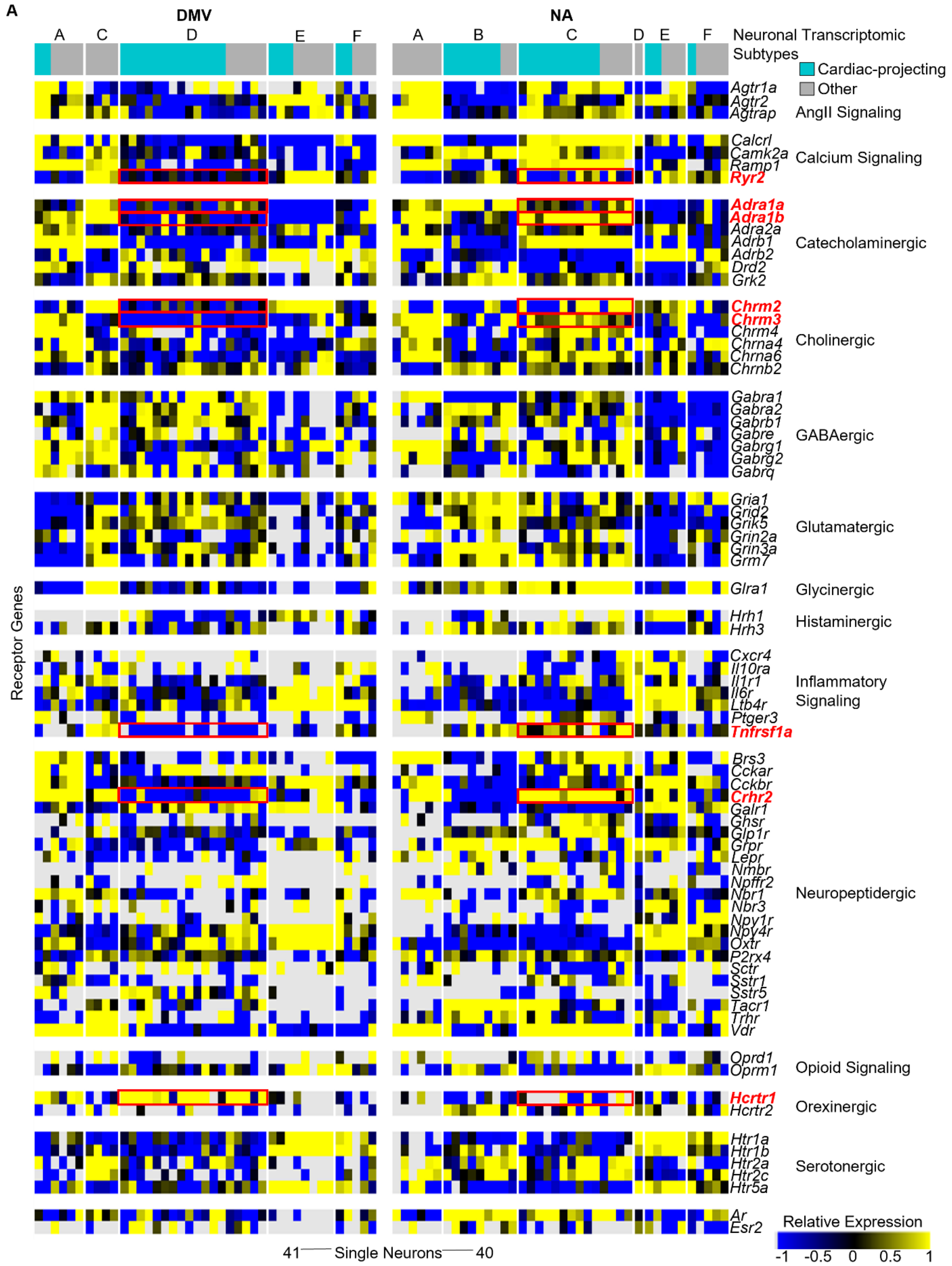

Figure S4. Distinctive co-expression of receptors in cardiac-projecting DMV neurons relative to other vagal motor neurons, related to Figure 5. (A) Normalized single neuron expression profiles of receptor genes annotated by brain region (DMV/NA), connectivity to the heart (cardiac-projecting or other), and neuronal transcriptomic subtype (states A-F). The genes are grouped by neuronal function relevant pathways. The red boxes indicate select differentially expressed genes between neuronal subtypes D and C, respectively (Tukey post-hoc $p < 0.05$).

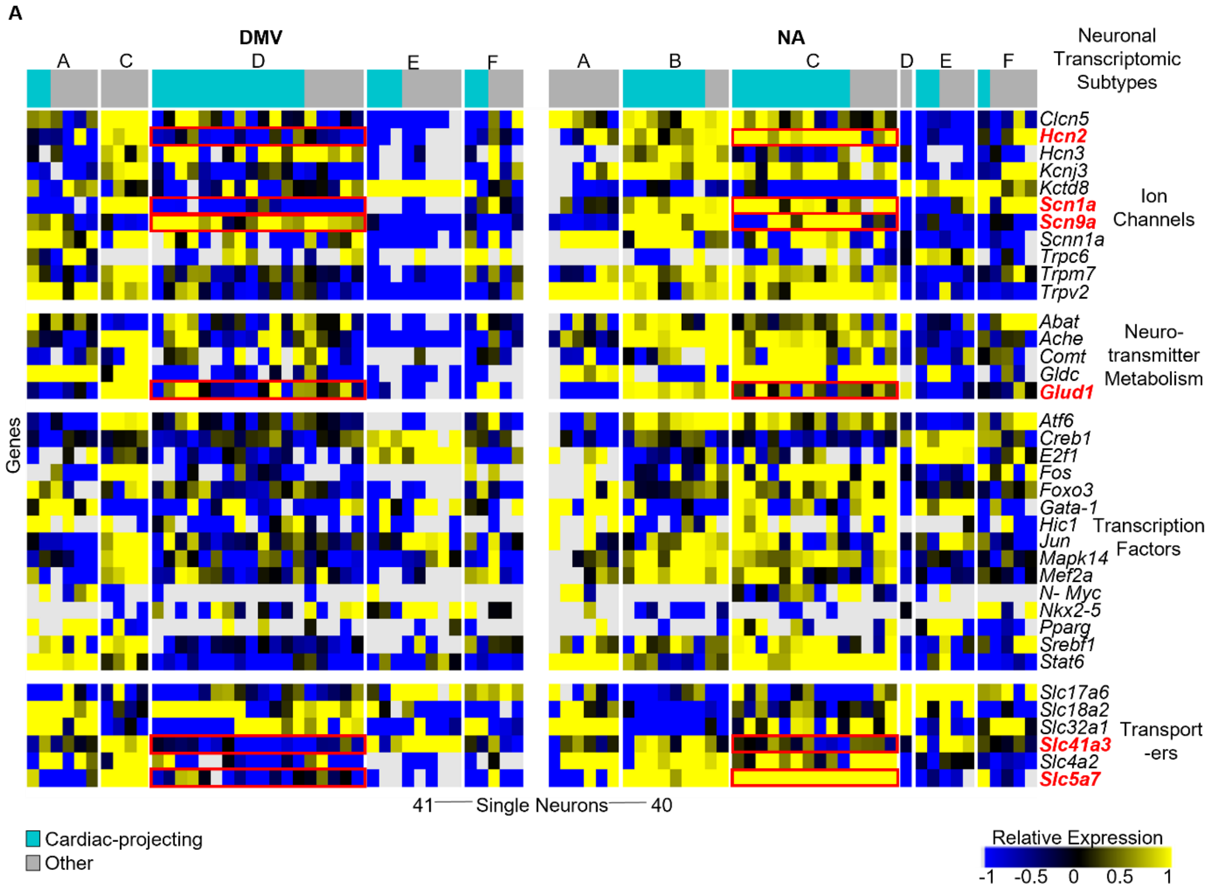

Figure S5. Distinctive co-expression of ion channels, neurotransmitter metabolism enzymes, transcription factors, and transporters in cardiac-projecting DMV neurons relative to other vagal motor neurons, related to Figure 5. (A) Normalized single neuron expression profiles of genes for ion channels, neurotransmitter metabolism enzymes, transcription factors, and transporters annotated by brain region (DMV/NA), connectivity to the heart (cardiac-projecting or other), and neuronal transcriptomic subtype (states A-F). The genes are grouped by neuronally relevant functions. The red boxes highlight three key genes that show (*Hcn3, Slc18a2, Slc5a7*) statistically significant differential expression between neuronal subtypes D and C, respectively (Tukey post-hoc $p < 0.05$).

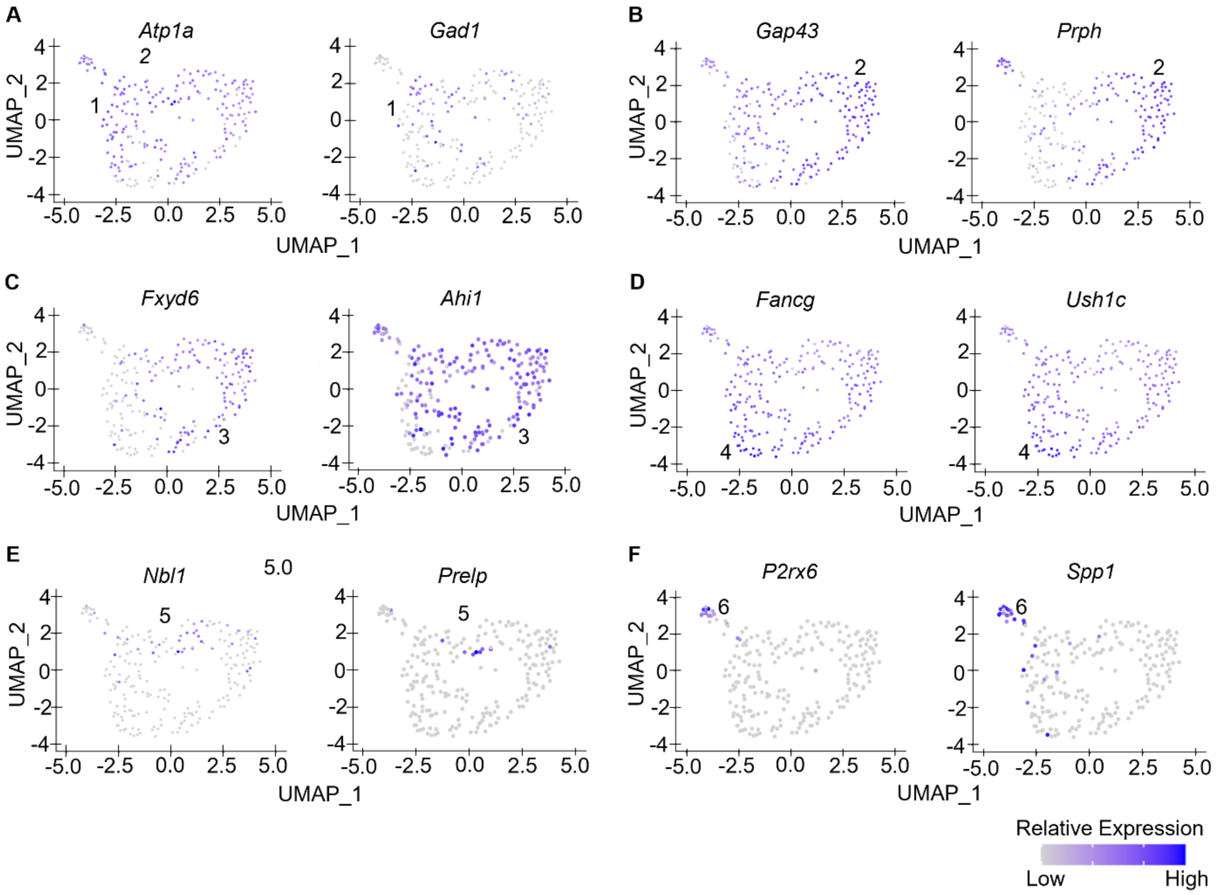

Figure S6. Top two transcriptomic markers per neuronal transcriptomic subtype, related to Figure 6. (A-F) Relative expression of top 2 transcriptomic markers for neuronal transcriptomic subtypes 1-6, respectively, overlaid over UMAP. $p < 0.05$.

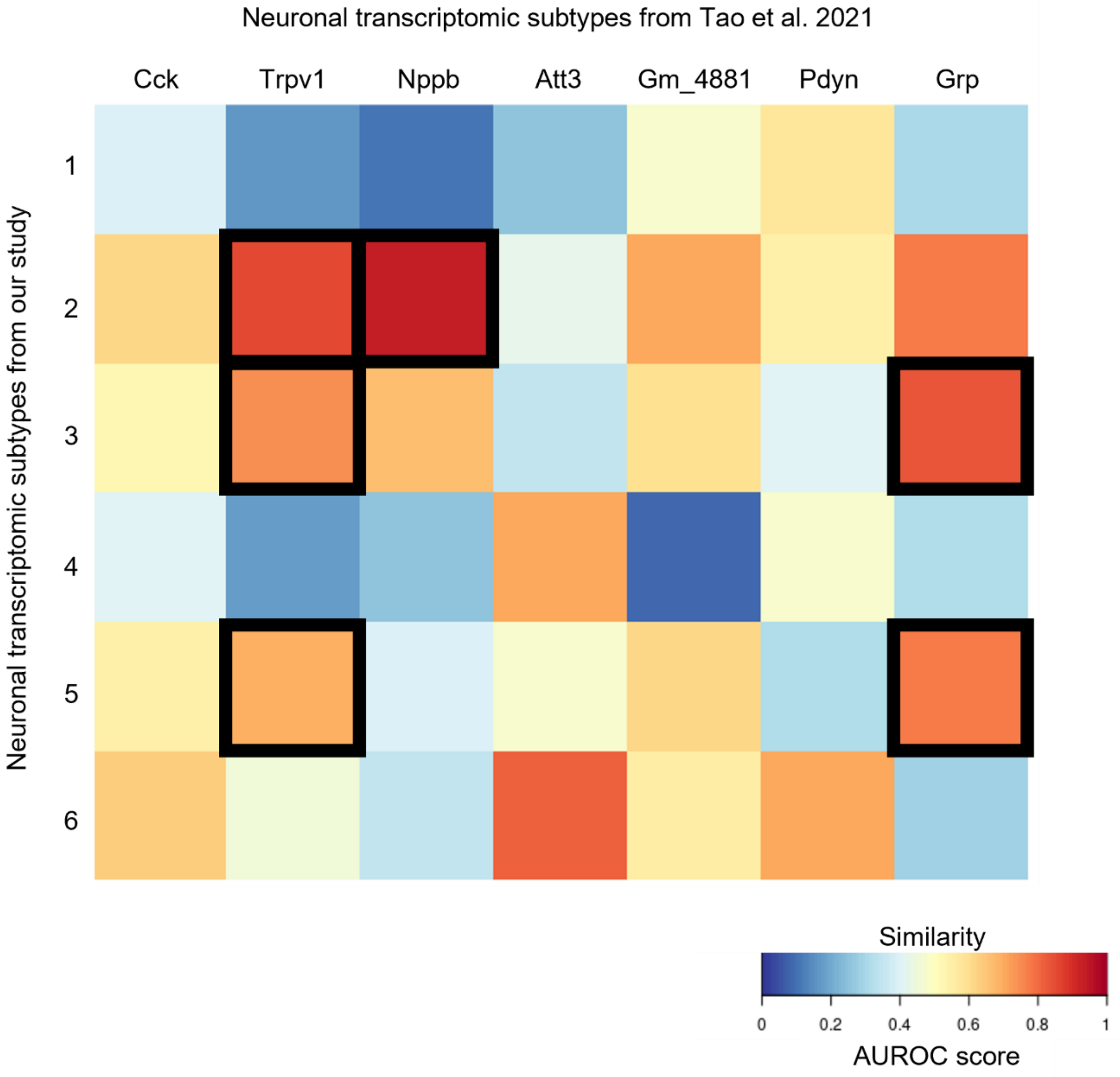

Figure S7. A comparison of DMV neuronal transcriptomic subtypes from the present study to those of Tao et al. 2021[5], related to Figure 6. Metaneighbor AUROC scores comparing the 6 neuronal transcriptomic subtypes from LCM-RNAseq data to the 8 neuronal transcriptomic subtypes from the Tao et al. 2021 single nucleus RNAseq data.

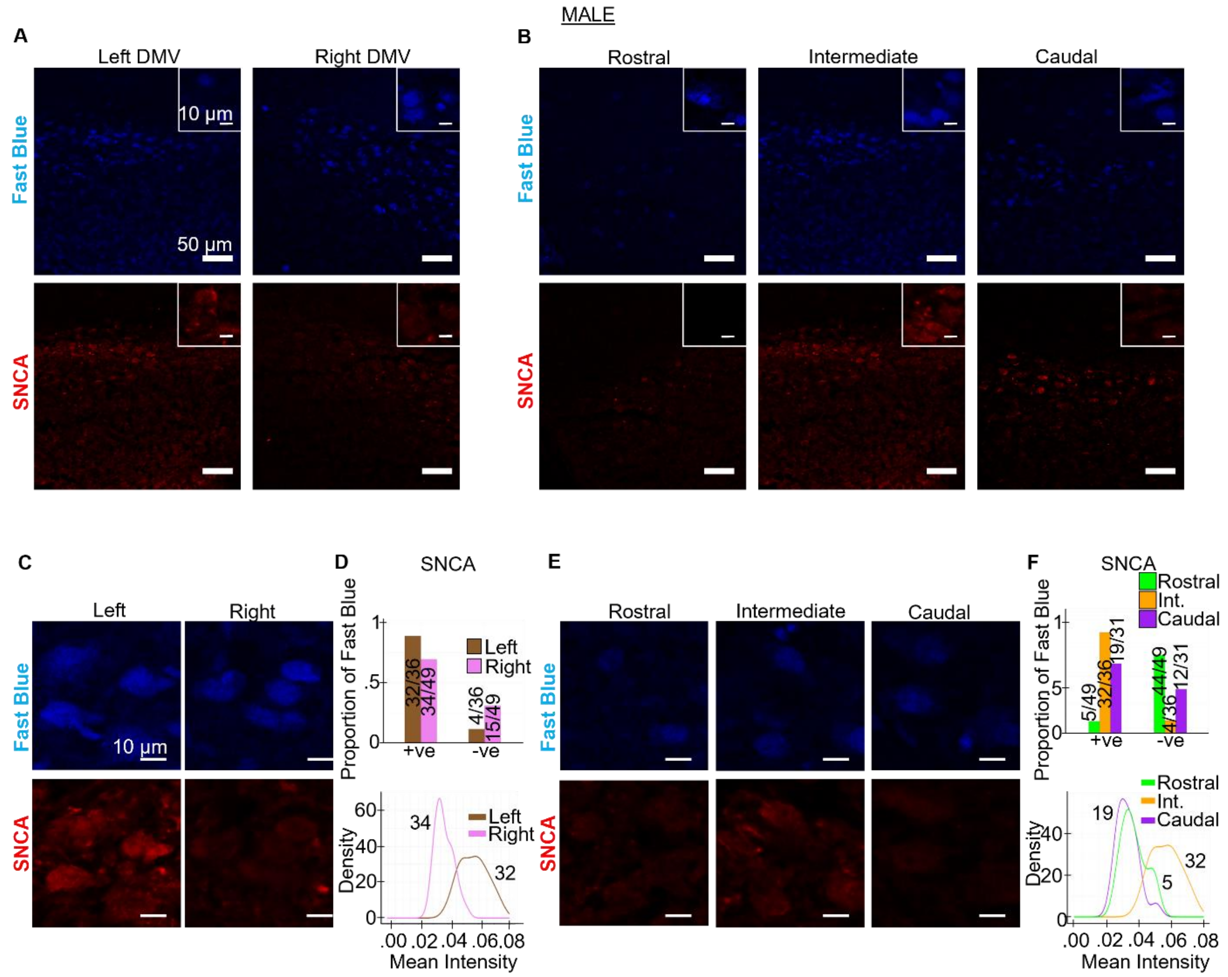

Figure S8. PACAP is selectively expressed by cardiac-projecting neurons of the intermediate left lateral DMV in male but not female rat, related to Figure 7. (A-D) Confocal immunofluorescence images of PACAP protein expression levels at 20X in cardiac-projecting neurons of (A, C) left and right DMV as well as (B, D) rostral, intermediate, and caudal left DMV in (A-B) male rat and (C-D) female rat as in (Figure 7). Zoomed in sets are at 400 % zoom. Representative images are shown from n = 3 sections.

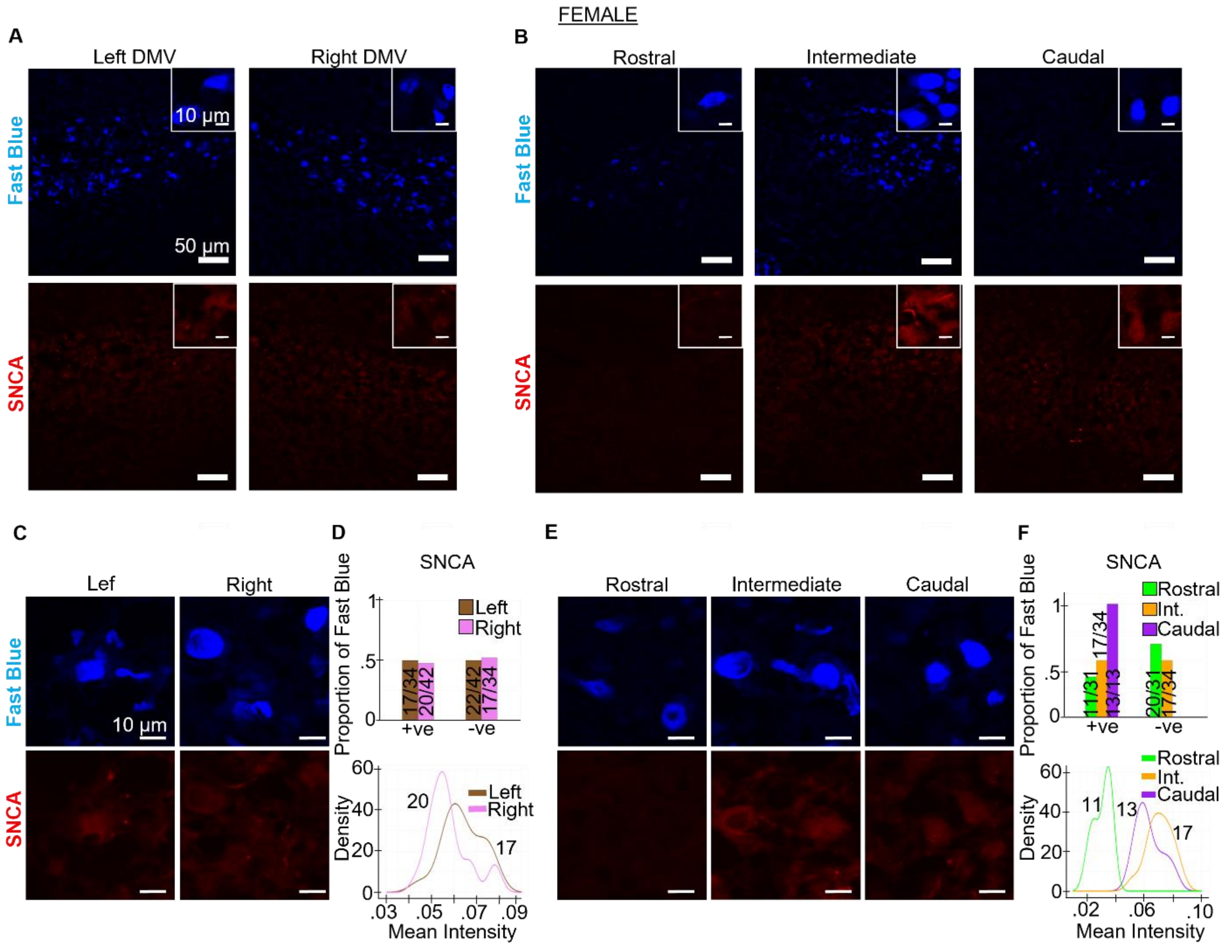

Figure S9. SNCA is selectively expressed by cardiac-projecting neurons of the left intermediate DMV in male rat, related to Figure 7. Confocal images at 20X of SNCA protein expression in tissue sections of (A) left and right DMV and (B) left rostral, intermediate, and caudal DMV at the level of Area Postrema in male rat. Representative images are shown from n = 3 sections. (C) Confocal images as in A at 400 % zoom. (D) Proportion of SNCA-positive and SNCA-negative cardiac-projecting neurons (top) and kernel density estimates of mean SNCA intensity values in cardiac-projecting neurons of the left and right DMV (bottom). Numbers indicate the number of SNCA-positive and SNCA-negative neurons in the corresponding region of a given section. (E) Confocal images as in B at 400 % zoom. (F) Proportion of SNCA-positive and SNCA-negative cardiac-projecting neurons (top) and kernel density estimates of mean SNCA intensity values in cardiac-projecting neurons of the rostral, intermediate, and caudal DMV (bottom). Numbers indicate the number of SNCA-positive and SNCA-negative neurons in the corresponding region of a given section.

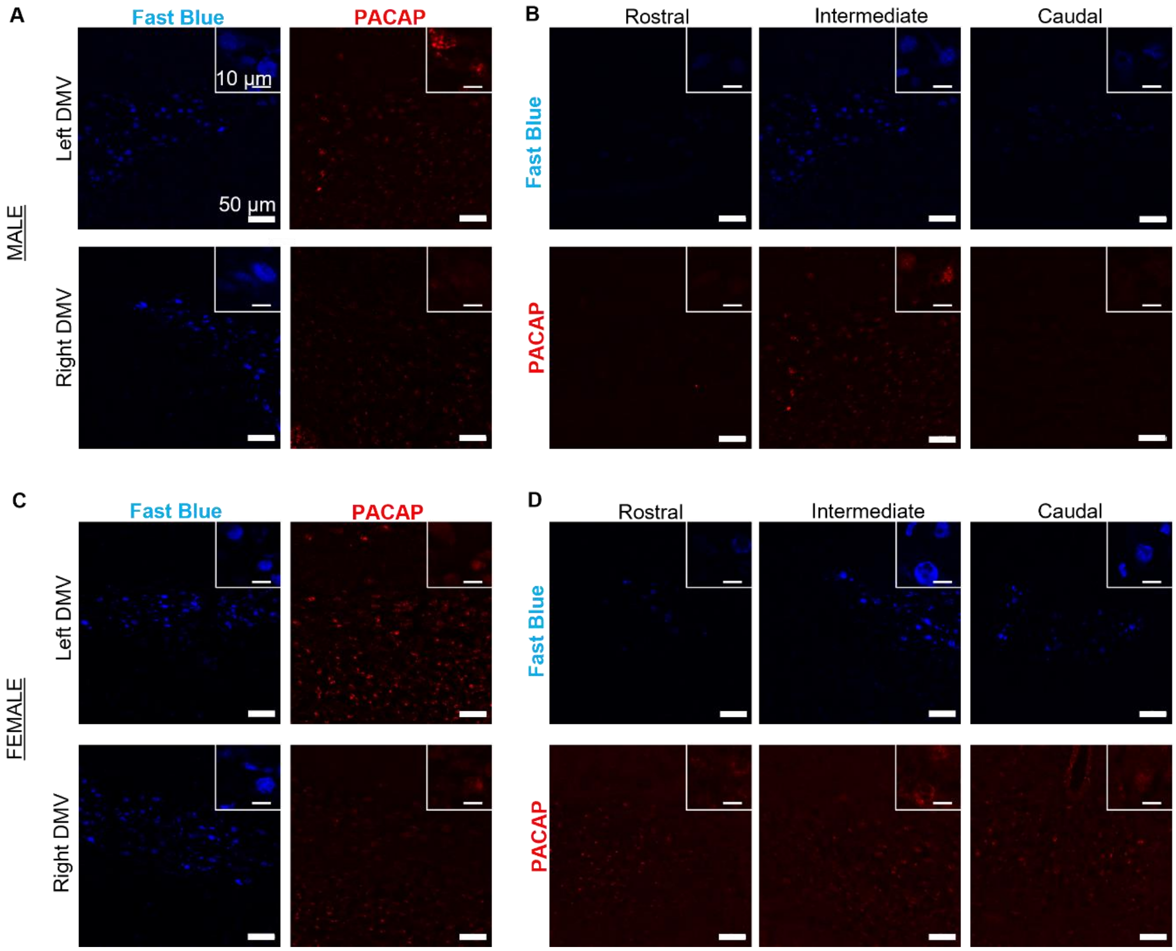

Figure S10. SNCA is selectively expressed by cardiac-projecting DMV neurons in female rat, related to Figure 7. Confocal images at 20X of SNCA protein expression in tissue sections of (A) left and right DMV and (B) left rostral, intermediate, and caudal DMV at the level of Area Postrema in female rat. Representative images are shown from n = 3 sections. (C) Confocal images as in A at 400 % zoom. (D) Proportion of SNCA-positive and SNCA-negative cardiac-projecting neurons (top) and kernel density estimates of mean SNCA intensity values in cardiac-projecting neurons of the left and right DMV (bottom). (E) Confocal images as in B at 400 % zoom. (F) Proportion of SNCA-positive and SNCA-negative cardiac-projecting neurons (top) and kernel density estimates of mean SNCA intensity values in cardiac-projecting neurons of the rostral, intermediate, and caudal DMV (bottom). Numbers indicate the number of SNCA-positive and SNCA-negative neurons in the corresponding region of a given section.
